# Imbalanced unfolded protein response signaling contributes to 1-deoxysphingolipid retinal toxicity

**DOI:** 10.1038/s41467-023-39775-w

**Published:** 2023-07-11

**Authors:** Jessica D. Rosarda, Sarah Giles, Sarah Harkins-Perry, Elizabeth A. Mills, Martin Friedlander, R. Luke Wiseman, Kevin T. Eade

**Affiliations:** 1 Department of Molecular Medicine, The Scripps Research Institute, La Jolla, CA 92037 USA; 2 Lowy Medical Research Institute, La Jolla, CA 92037 USA

**Keywords:** Mechanisms of disease, Stress signalling, Protein quality control, Retinal diseases

## Abstract

The accumulation of atypical, cytotoxic 1-deoxysphingolipids (1-dSLs) has been linked to retinal diseases such as diabetic retinopathy and Macular Telangiectasia Type 2. However, the molecular mechanisms by which 1-dSLs induce toxicity in retinal cells remain poorly understood. Here, we integrate bulk and single-nucleus RNA-sequencing to define biological pathways that modulate 1-dSL toxicity in human retinal organoids. Our results demonstrate that 1-dSLs differentially activate signaling arms of the unfolded protein response (UPR) in photoreceptor cells and Müller glia. Using a combination of pharmacologic activators and inhibitors, we show that sustained PERK signaling through the integrated stress response (ISR) and deficiencies in signaling through the protective ATF6 arm of the UPR are implicated in 1-dSL-induced photoreceptor toxicity. Further, we demonstrate that pharmacologic activation of ATF6 mitigates 1-dSL toxicity without impacting PERK/ISR signaling. Collectively, our results identify new opportunities to intervene in 1-dSL linked diseases through targeting different arms of the UPR.

## Abstract (web summary)

The accumulation of cytotoxic deoxysphingolipids causes retinopathies through unknown mechanisms. Here the authors use retinal organoids to show that photoreceptor toxicity is mediated by unfolded protein response signaling.

## Introduction

Sphingolipids (SLs) are a class of membrane lipids central to the synthesis of both structural lipids including ceramides, sphingomyelin, and glycosphingolipids, and bioactive signaling lipids such as sphingosine-1-phosphate^1^. In the cell, SLs are synthesized de novo through the condensation of fatty acyl chains to serine, which provides a hydroxyl moiety to which different head groups can be attached. However, during SL synthesis, alanine can be substituted for serine to generate 1-deoxysphingolipids (1-dSLs) that lack the hydroxyl required for lipid functionalization. While the synthesis of 1-dSLs is naturally low, the accumulation of 1-dSLs is cytotoxic^2^ and associated with the pathogenesis of numerous retinopathies and neuropathies including type I and type II diabetes, Macular Telangiectasia (MacTel), and hereditary sensory neuropathy type 1 (HSAN1)^3–6^.

Gain of function mutations in the first enzyme of the SL biosynthetic pathway, serine palmitoyltransferase (SPT), lead to accumulation of 1-dSL and subsequent neurotoxicity in HSAN1 and retinal degeneration in MacTel^3,4^. While pathologic SPT mutations are very rare (only a few hundred cases worldwide), MacTel is a more common disease with a prevalence reported up to ~1:1000^7^. MacTel has an extremely heterogenous genetic architecture that converges on a shared metabolic phenotype involving reductions in circulating levels of serine^3^. This reduction in serine drives an elevation of 1-dSLs that correlates with the severity of retinal degeneration. The accumulation of 1-dSLs has also been associated with the onset of peripheral neuropathy and retinopathy in diabetes, where elevated levels are a strong predictive risk factor for type II diabetes^5,8,9^ and correlate with peripheral neuropathy implicated in type I diabetes^6^.

Intracellular accumulation of 1-dSLs impacts a variety of cellular processes including mitochondrial function^10^, lipid body formation^11^, protein folding^12^, autophagy^13^, cytoskeletal reorganization^14^, endocytosis^15^, calcium handling^16^, and ER stress^11,17^. Furthermore, 1-dSLs have been suggested to induce cell death through atypical cell death programs in fibroblast, liver, and neuroblastoma cell lines^2^. 1-dSL toxicity can also vary between the same cells cultured under different conditions, underscoring the sensitivity of 1-dSL toxicity to multiple biological factors^15^. A meta-analysis suggests that neuronal cells are more sensitive to 1-dSLs than other cell types^18^, consistent with the pathogenic neuropathy observed in response to 1-dSL accumulation in diseases such as HSAN1 and diabetes. However, despite the clear link between 1-dSLs and cytotoxicity, the pathologic mechanism of 1-dSL toxicity in complex tissues such as the retina remains unclear.

**Fig. 1:**
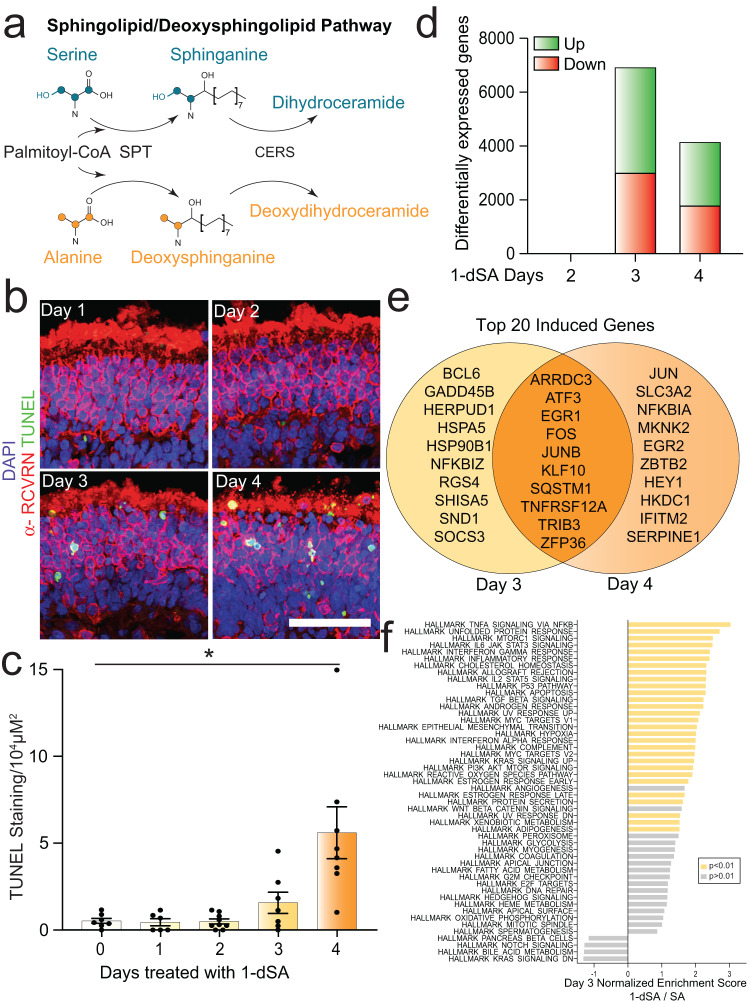
1-dSL-induced transcriptional remodeling precedes cell death. **a** Schematic of the 1-deoxysphingolipid (1-dSL) biosynthesis pathway. SPT serine palmitoyltransferase; CERS ceramide synthase. **b** Representative immunostaining of the photoreceptor marker α-RCVRN (red), the cell death marker TUNEL stain (green), and nuclear DAPI stain (blue) in retinal organoids treated with 1-dSA (1 µM) for 1–4 days. Scale bar is 25 µm. **c** Quantification of TUNEL staining from images as shown in **b** presented as mean ± SEM. Each dot represents a biologically independent RO tested concurrently. *p* = 0.0411 for a Welch ANOVA with Dunnett T3 test for multiple comparisons to Day 0. *p < 0.05. **d** Quantification of differentially expressed mRNA transcripts measured by RNAseq of ROs (*n* = 5 biologically independent replicates of seven pooled ROs, per condition, per day) treated with 1-dSA (1 µM) for 2, 3, and 4 days relative to control-treated ROs. **e** Venn diagram showing the distinct and shared transcripts among the top 20 mRNA transcripts with the highest *p* values showing increased expression from RNAseq data presented in **d**. **f** Enrichment of MsigDB Hallmark pathways in RNAseq data of ROs treated with 1-dSA relative to SA for 3 days (*n* = 5 biologically independent replicates of seven pooled ROs, per condition). Pathways with enrichment of *p* adj <0.01 are highlighted in yellow. Source data are provided as a Source Data file.

Here, we sought to define a general pathologic mechanism for 1-dSL-induced retinopathy using human retinal organoids (ROs). ROs derived from human induced pluripotent stem cells (iPSCs) are functional retinal tissues that recapitulate key aspects of the cytoarchitecture, cellular diversity, and function of the human retina^19–21^. We previously found that elevation of 1-dSL concentrations caused apoptosis in RO photoreceptors and could be rescued through treatment with drugs that directly alter lipid metabolism, including the dyslipidemia drug, fenofibrate^3^. The cytotoxic effect of 1-dSLs is largely attributed to the accumulation of the metabolite 1-deoxydihydroceramide (1-dDHCer)^3,12^, which is synthesized from 1-deoxysphinganine (1-dSA) by ceramide synthases (CERSs) in the endoplasmic reticulum (ER) (Fig. 1a)^22^. Subcellular localization studies using labeled 1-dSA in fibroblasts show that 1-dSA accumulates in the ER, Golgi, and mitochondria resulting in compromised organelle structure^10,23,24^. Further, 1-dSA accumulation induces activation of ER stress-responsive genes including *XBP1s* and the pro-apoptotic transcription factor *DDIT3/CHOP* in other cell types^10,25^. This suggests that ER stress and impaired ER regulation could contribute to the pathogenic mechanism of 1-dSA-induced retinal toxicity.

The unfolded protein response (UPR) is the primary stress-responsive signaling pathway responsible for regulating cellular physiology during ER stress and comprises three signaling pathways activated downstream of the ER stress-sensing proteins IRE1, PERK, and ATF6^26,27^. In response to ER stress, these pathways induce translational and transcriptional signaling that function to both alleviate the ER stress and promote adaptive remodeling of diverse biological pathways involved in cellular functions including protein secretion, lipid synthesis, and calcium regulation^27,28^. However, chronic UPR activation induces maladaptive signaling, primarily through the PERK-dependent upregulation of pro-apoptotic factors such as *DDIT3/CHOP*, that induce cell death in response to sustained, unresolvable ER stress^27,29^. Dysregulated UPR signaling is implicated in the onset and pathogenesis of numerous diseases, including many retinal diseases^30,31^. Deficiencies in ATF6 signaling lead to impaired cone photoreceptor development implicated in the disease achromatopsia^28,32^. Further, dysregulated signaling through all three UPR pathways is associated with retinal degeneration in diseases such as retinitis pigmentosa^31–34^. This suggests that the retina, and specifically photoreceptor cells within the retina, are highly sensitive to imbalances in UPR signaling.

In this study, we used bulk and single-nucleus RNAseq of mature human ROs to define pathologic mechanisms that contribute to 1-dSL-induced toxicity. We found that 1-dSA treatment induced activation of UPR signaling pathways in photoreceptors and Müller glia, while only minimally impacting gene expression in other cell types. Intriguingly, adaptive IRE1/XBP1s and ATF6 signaling are only observed transiently at early stages of 1-dSA treatment, with activity decreasing at later stages when photoreceptor death is prevalent. In contrast, signaling through the pro-apoptotic PERK arm of the UPR and the related integrated stress response (ISR)^35^ is observed in photoreceptors throughout the 1-dSA treatment paradigm. This suggests that deficient signaling through the adaptive IRE1/XBP1s and ATF6 UPR pathways and sustained signaling through the pro-apoptotic PERK/ISR UPR pathway contribute to 1-dSL-induced retinal toxicity. Consistent with this, pharmacologic inhibition of PERK/ISR signaling reduces 1-dSL toxicity in retinal organoids by suppressing the expression of pro-apoptotic and inflammatory genes. In contrast, pharmacologic inhibition of ATF6 activity increases 1-dSL toxicity, while enhancing activation of this UPR signaling pathway mitigates 1-dSL-induced photoreceptor cell death. However, the protection afforded by pharmacologic ATF6 activation does not correspond to reductions in PERK/ISR signaling, indicating that the relative activity of these two UPR pathways is an important determinant in dictating 1-dSL toxicity in retinal organoids. Instead, we show that ATF6 protection is likely mediated through the regulation of neuroprotective factors such as MANF. Collectively, our results demonstrate that imbalanced signaling through the UPR, most notably the PERK and ATF6 signaling arms, is a key pathogenic mechanism in 1-dSA-induced retinal toxicity. Further, our results indicate that pharmacologic interventions which correct imbalanced UPR signaling present new opportunities to therapeutically attenuate the retinal degeneration associated with 1-dSL toxicity in diseases such as MacTel or diabetic retinopathy.

## Results

### Treatment with 1-dSA induces time-dependent toxicity in retinal organoids

We sought to define the pathologic mechanisms that contribute to 1-dSL-induced toxicity in ROs. We initially monitored apoptosis in ROs treated with 1-dSA (18:0) for 1–4 days using TUNEL staining. No TUNEL staining was observed following 1–2 days of treatment, with a modest increase following 3 days of treatment (Fig. 1b, c). Retinal cell death was prominently observed on day 4. The majority of TUNEL staining was observed in the outer nuclear layer (ONL), which primarily consists of photoreceptors (Fig. S1a). This is consistent with the previously observed sensitivity of photoreceptors to 1-dSA^3,36^.

To define pathways involved in the initial steps of 1-dSA toxicity, we performed whole transcriptome RNAseq on ROs treated with 1-dSA for 2, 3, or 4 days. We observed only 1 differentially expressed gene (DEG) following 2 days of treatment, with substantially higher DEGs observed following 3 or 4 days of treatment (Fig. 1d, Supp. Data 1–3). The highest number of DEGs were observed following 3 days of treatment, corresponding to the modest increase in toxicity observed at this time point (Fig. 1b, c). The transcriptional response observed upon treatment of ROs with 1-dSA for 3 days was distinct from that observed in ROs treated with the non-toxic SL, sphinganine (Fig. S1b, c, Supp. Data 2), indicating that the observed transcriptional response was specific to deoxy-derived SLs rather than a broader response to exogenous lipids. DEGs observed in ROs treated for 3 or 4 days with 1-dSA showed significant overlap, although many genes were also found to be differentially expressed between these two time points (Fig. 1e, Fig. S1d). Gene set enrichment analysis (GSEA) demonstrated enrichment of pathologic pathways involved in inflammation, apoptosis, and UPR signaling at both 3 and 4 days of treatment (Fig. 1f, Fig. S1e). This suggests an important role for these biological pathways in 1-dSA retinal toxicity.

### Photoreceptors and Müller glia are selectively sensitive to 1-dSA in ROs

**Fig. 2:**
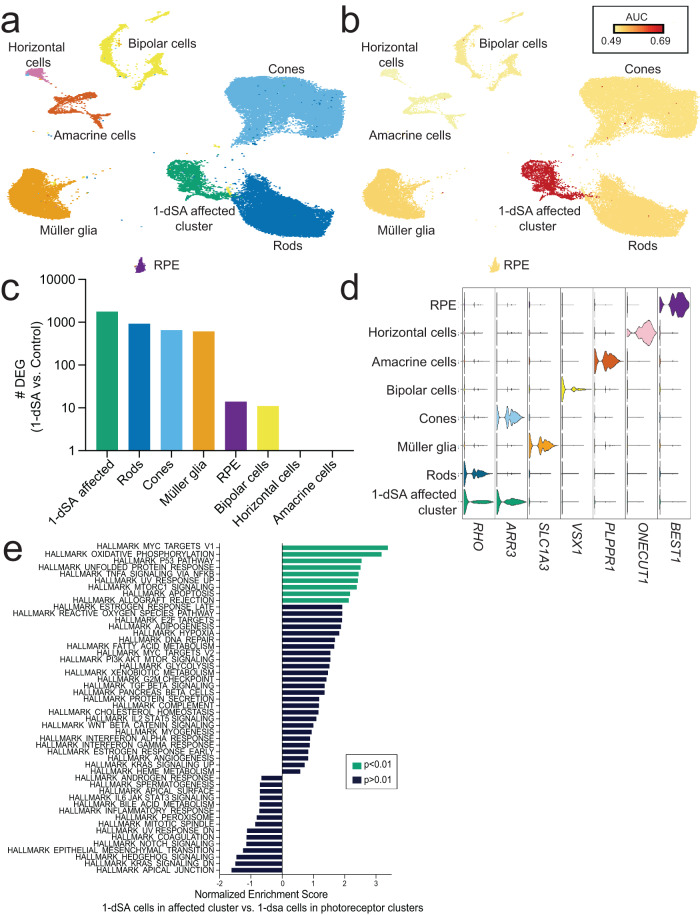
Photoreceptors and Müller glia show a transcriptional response to 1-dSA. **a** UMAP of mature retinal cell types generated by snRNAseq from ROs treated with control or 1-dSA (1 µM) for 3 days colored by cluster (*n* = 2 biologically independent replicates of seven pooled ROs per condition). Clusters were assigned a retinal cell type based on previously defined markers. **b** Augur-generated UMAP of mature retinal cell responsiveness to 1-dSA treatment, measured as how accurately 1-dSA or control treatment could be predicted for each cell and reported as the area under the receiver operating characteristic curve (AUC). The AUC ranges from 0 to 1, where an AUC of 0.5 corresponds to the accuracy of a random classifier and an AUC of 1 represents perfect classification. **c**. Bar graph showing the total number of differentially expressed genes (DEGs) between control and 1-dSA (1 µM; 3 days) organoids for each mature retinal cell cluster identified in **a**. **d** Violin plot of mature retinal cell type marker expression by clusters, as defined in **a**. **e** Enrichment plot of differentially expressed pathways between 1-dSA treated cells in the affected cluster compared to 1-dSA treated cells in the combined rod and cone clusters. Pathways enriched in the 1-dSA affected cluster (*p* < 0.01) are highlighted in green. Source data are provided as a Source Data file.

To gain further insights into the cell type-specific mechanisms of 1-dSA-induced toxicity, we performed single-nucleus RNAseq (snRNAseq) on iPSC-derived ROs treated with or without 1-dSA for 3 days—a timepoint when substantial transcriptional remodeling is first observed (Fig. 1d). Integrated clustering of control and 1-dSA-treated ROs showed overlapping groups of mature retinal cell types including rod and cone photoreceptors, Müller glia, accessory cell types, and immature and progenitor cells (Fig. 2a, Fig. S2a). To determine which mature retinal cell types were strongly impacted by 1-dSA treatment, we applied Augur, a computational method that ranks the responsiveness of cell types to perturbations without bias to the number of cells in a cluster^37^. The Augur-generated UMAP identified a  cluster with the most substantial transcriptional shift, herein is referred to as the 1-dSA affected cluster (Fig. 2b). This population was poorly represented in control-treated organoids but substantially increased in response to 1-dSA (Fig. S2b), which suggested that this cluster represented a strongly impacted subpopulation of retinal cells. While the 1-dSA affected cluster exhibited the most profound transcriptional shift following treatment with 1-dSA, photoreceptors and Muller glia also showed pronounced transcriptomic changes between 1-dSA and control treatments (Fig. 2b).

Consistent with this, 1-dSA substantially increased the number of DEGs in the 1-dSA affected cluster, Müller glia, as well as cone and rod photoreceptors, with minimal effects on other retinal cell types (Fig. 2c, Supp. Data 4). GSEA analysis of differentially expressed genes in 1-dSA treated populations of Müller glia and cone/rod photoreceptors showed that all three of these cell types demonstrated enrichment of pathways involved in inflammation, apoptosis, and UPR signaling relative to control populations (Fig. S2c–e). A similar set of enriched pathways were observed in the bulk RNAseq of 1-dSA-treated ROs (Fig. 1f), indicating that the majority of transcriptional changes in retinal organoids can be attributed to these cellular populations.

In order to determine which retinal cells comprise the 1-dSA-affected cluster, we assessed the presence of cell type-specific markers within the cluster^21^. Cells in the affected cluster expressed markers of cones (e.g., *ARR3*) and rods (e.g., *RHO*), but not markers of other retinal cell types (Fig. 2d, Fig. S2f). The increase in cells observed in the 1-dSA affected cluster also corresponded with reductions in cone photoreceptors (Fig. S2g), further indicating that this population reflects an altered state of photoreceptor cells. A comparison of gene expression between the 1-dSA affected cluster, and the rod and cone clusters within the 1-dSA treatment shows further enrichment of multiple stress-responsive pathways including oxidative phosphorylation, P53-mediated apoptosis, and the UPR (Fig. 2e). This suggests that cells within the 1-dSA affected cluster are likely photoreceptors primed for death. This is consistent with TUNEL staining showing the majority of cell death occurring in the photoreceptors of the RO ONL (Fig. S1a).

### The three arms of the UPR are differentially activated in retinal cell types

GSEA of our bulk RNAseq identified the UPR as a prominent stress pathway activated in retinal organoids treated with 1-dSA for both 3 (Fig. 1f) or 4 days (Fig. S1e). We similarly observed significant upregulation of the UPR in photoreceptors and Müller glia (Fig. 2e, S3a, b), the cell types most impacted by 1-dSA treatment. Based on the prominent and timely activation of the UPR in 1-dSA treated ROs, and previous studies that have localized the conversion of 1-dSA to the toxic species 1-dDHCer by CERS in the ER, we hypothesized that the ER stress-responsive UPR plays an important role in 1-dSA mediated toxicity.

**Fig. 3:**
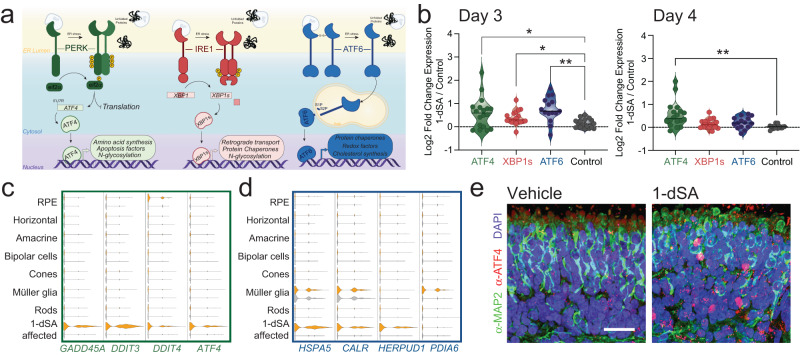
1-dSA differentially activates unfolded protein response signaling pathways in specific retinal cell types. **a** Illustration showing the transcriptional and translational remodeling which occurs downstream of PERK, IRE1, and ATF6 activation. **b** Quantification of bulk RNAseq fold changes of gene targets of the UPR-induced transcription factors ATF4, XBP1s, and ATF6 in ROs (*n* = 5 biologically independent replicates of seven pooled ROs per condition) treated with 1-dSA (1 µM) relative to control for 3 or 4 days. Day 3 ATF4 vs. Control *p* = 0.0353, XBP1s vs. control *p* = 0.0185, ATF6 vs. control *p* = 0.0017, and Day 4 ATF4 vs. Control *p* = 0.0032 for Welch ANOVA test with Dunnett T3 corrections for multiple comparisons to control gene set. *p < 0.05, **p < 0.01. **c** Violin plot of gene targets of ATF4 in mature cells from snRNAseq dataset (*n* = 2 biologically independent replicates of seven pooled ROs per condition) separated by cluster and treatment. 1-dSA-treated organoids (3 days; 1 µM) are in orange; control organoids are in gray. **d** Violin plot of gene targets of ATF6 in snRNAseq dataset (*n* = 2 biologically independent replicates of seven pooled ROs per condition) separated by cluster and treatment. 1-dSA treated organoids (3 days; 1 µM) are in orange; control organoids are in gray. **e** Immunostaining of the pan-neuronal marker α-MAP2 (green), α-ATF4 (red), and nuclear stain DAPI (blue) in ROs treated with control or 1-dSA (1 µM) for 4 days. Scale bar is 25 µm. Source data are provided as a Source Data file.

The UPR comprises three signaling pathways activated downstream of the ER stress-sensing proteins PERK, IRE1, and ATF6 (Fig. 3a)^27^. In response to ER stress, these pathways promote transcriptional remodeling through the activation of the UPR-associated transcription factors ATF4, XBP1s, and cleaved ATF6, respectively^26,27^. We used sets of genes differentially regulated downstream of these three UPR signaling pathways^38^ to define the relative activity of PERK/ATF4, IRE1/XBP1s, and ATF6 signaling in ROs treated with 1-dSA for 3 days. Bulk RNAseq showed that 1-dSA induced expression of target genes associated with all three UPR arms, indicating that all three UPR signaling pathways are activated at this timepoint (Fig. 3b, Fig. S3c). Further, snRNAseq showed increased expression of target genes regulated by PERK/ATF4 (e.g., *DDIT3*, *ATF4*) and ATF6 (e.g., *CALR, HSPA5, HERPUD1*) in the 1-dSA affected cluster following three days of treatment (Fig. 3c, d). The canonical XBP1s target *DNAJB9* showed the highest levels of expression in the 1-dSA-affected cluster (Fig. S3d); however, other XBP1s target genes were overall poorly detected and could not be used to define patterns of IRE1 activity in individual retinal cell types (Fig. S3e). This suggests that all three UPR arms are activated in the 1-dSA-affected cluster under these conditions. In contrast, Müller glia showed basally higher expression of proteostasis factors regulated by ATF6 during ER stress conditions (e.g., *CALR, HSPA5*)^38,39^ and also showed 1-dSA-induced increases in the expression of other ATF6 targets such as *PDIA6* (Fig. 3d). PERK/ATF4 target genes were not induced by 1-dSA in these cells (Fig. 3c). No other retinal cell type showed significant activation of any UPR signaling pathways in our snRNAseq data. These results indicate that a distinct UPR signature was preferentially induced in the 1-dSA-affected cluster of photoreceptors and Müller glia in ROs treated with 1-dSA for 3 days.

Intriguingly, while ROs treated for 3 days with 1-dSA showed activation of genes regulated by PERK/ATF4, IRE1/XBP1s, and ATF6, RNAseq data from ROs treated for 4 days with 1-dSA showed preferential activation of the PERK/ATF4 signaling arm of the UPR (Fig. 3b). Despite seeing increased expression of select IRE1/XBP1s and ATF6 target genes, the majority of targets associated with these pathways were not induced at day 4 (Supp. Data 3). This suggests that the IRE1/XBP1s and ATF6 pathways are not robustly activated at this timepoint. We confirmed prominent increases of ATF4 protein expression following four days of 1-dSA treatment by immunostaining (Fig. 3e). Collectively, our results indicate that all three arms of the UPR are activated at day 3 of 1-dSA treatment, a timepoint before significant RO toxicity is observed (Fig. 1b, c). However, only PERK/ATF4 signaling persists at day 4 when higher levels of TUNEL staining are observed.

### PERK signaling promotes 1-dSA toxicity in ROs

**Fig. 4:**
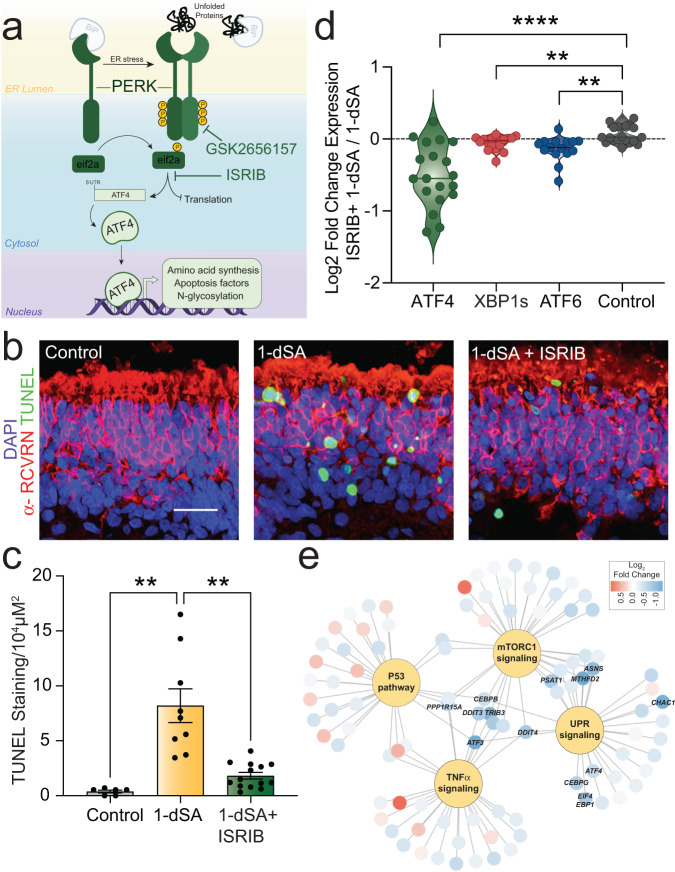
PERK/ISR signaling contributes to 1-dSA-induced retinal toxicity. **a** Illustration showing PERK signaling induced by ER stress. GSK2656157 inhibits PERK autophosphorylation and ISRIB inhibits signaling downstream of eIF2α phosphorylation. **b**, **c** Representative images and quantification of TUNEL staining of organoids treated for 4 days with 1-dSA (1 µM) in the presence or absence of the PERK/ISR signaling inhibitor ISRIB (200 nM). **b** Immunostaining of the photoreceptor marker α-RCVRN (red), the cell death marker TUNEL stain (green), and nuclear DAPI stain (blue). Scale bar is 25 µm. **c** Quantification presented as mean ± SEM. Each dot represents biologically independent, individual ROs tested concurrently. Control vs. 1-dSA *p* = 0.0019, 1-dSA vs. 1-dSA vs. ISRIB *p* = 0.0055 for Welch ANOVA test with Dunnett T3 corrections for multiple comparisons to 1-dSA. **p < 0.01. **d** Quantification of RNAseq fold changes of gene targets of the UPR-induced transcription factors ATF4, XBP1s, and ATF6 in ROs treated with 1-dSA (1 µM) in the presence or absence of ISRIB (200 nM) for 4 days (*n* = 5 replicates of seven pooled ROs per condition). ATF4 vs. control *p* < 0.0001, XBP1s vs. Control *p* = 0.0041, ATF6 vs. Control *p* = 0.0020 for Welch ANOVA test with Dunnett T3 corrections for multiple comparisons to control. **** **p < 0.01, *p* < 0.0001. **e** Network graph showing shared transcripts and associated fold changes of selected Hallmark gene set pathways significantly altered by treatment with 1-dSA. Source data are provided as a Source Data file.

Chronic or hyperactive PERK/ATF4 signaling induces apoptosis in multiple models through increased expression of pro-apoptotic factors such as *DDIT3/CHOP*^29,40,41^. Our observation that PERK/ATF4 target genes show persistent activation in 1-dSA-treated ROs suggests that hyperactive PERK signaling could contribute to the observed toxicity in this model. We monitored toxicity in ROs treated with 1-dSA in the presence or absence of two mechanistically distinct PERK signaling inhibitors; GSK2656157 and ISRIB. GSK2656157 is a PERK kinase inhibitor that blocks PERK autophosphorylation required for the activation of this UPR signaling pathway (Fig. 4a)^42^. Alternatively, ISRIB inhibits PERK signaling downstream of eIF2α phosphorylation^43–45^. Thus, unlike GSK2656157, ISRIB can also inhibit eIF2α phosphorylation and downstream signaling induced by other eIF2α kinases (e.g., GCN2, HRI, PKR) comprising the integrated stress response (ISR)^35,45^. Co-treatment with either ISRIB (Fig. 4b, c) or GSK2656157 (Fig. S4a, b) reduced TUNEL staining in ROs treated with 1-dSA, indicating that PERK signaling contributes to 1-dSA-induced toxicity in this model. ISRIB showed a stronger reduction in toxicity, as compared to GSK2656157, potentially indicating a role for other ISR kinases in this toxicity. Both compounds inhibited ATF4 target gene expression in 1-dSA-treated ROs, confirming their activity (Fig. 4d, S4c, Supp. Data 5). We also confirmed that ISRIB suppressed the expression and nuclear localization of ATF4 in 1-dSA-treated organoids (Fig. S4d). These results indicate that PERK/ISR signaling is an important contributor to photoreceptor death in 1-dSA-treated ROs.

To further define the involvement of PERK signaling in this process, we performed bulk RNAseq on ROs treated with 1-dSA and/or ISRIB for 4 days. Co-treatment of 1-dSA with ISRIB reduced the expression of PERK/ATF4 target genes in this model. ISRIB modestly, but significantly, decreased the expression of genes regulated by other arms of the UPR (Fig. 4d) to a lesser extent than established direct targets of ATF4. As ATF4 regulates the expression of ATF6, and ATF6 regulates XBP1 expression, this likely reflects a feedback loop between the PERK/ISR and other arms of the UPR^38,46,47^. GSEA shows that ISRIB co-treatment also reduced expression of genes involved in P53-mediated apoptosis, TNFα mediated inflammation, and mTORC1 activation (Fig. S4e)—three pathways upregulated by 1-dSA in Müller glia and photoreceptor clusters (Fig. 2e, Fig. S2c–e). However, these reductions can be largely attributed to the reduced expression of ATF4-regulated genes including *DDIT3*, *ATF3*, and *PPP1R15A* (Fig. 4e, Fig. S4f). This suggests that ISRIB reduces inflammation, apoptosis, and mTORC1 signaling by suppressing the expression of ATF4 targets involved in these pathways rather than broadly impacting inflammatory, apoptotic, or metabolic signaling. Collectively, these results indicate that pharmacologic inhibition of PERK/ISR signaling selectively impacts the transcriptional response to 1-dSA in ROs predominantly by suppressing ATF4 activity.

The above results suggest pharmacologic inhibition of PERK/ISR signaling as a potential strategy to mitigate 1-dSA-induced toxicity. However, PERK/ISR signaling is also implicated in the regulation of amino acids, including serine^48,49^. Notably, PERK/ISR activation induces the ATF4-mediated expression of key serine biosynthesis enzymes including *PSAT1* and *PHGDH*. Decreased activity of these enzymes reduces serine availability and increases cellular production of 1-dSA and toxic dSLs^3,11,50^. This suggests that pharmacologic inhibition of PERK/ISR signaling could exacerbate 1-dSA production by suppressing serine biosynthesis. Consistent with this, ISRIB reduced the expression of serine biosynthesis genes in ROs both in the absence and presence of 1-dSA (Fig. S4g, h). Thus, while PERK/ISR inhibition blocks 1-dSA toxicity in ROs, it may exacerbate the production of toxic 1-dSLs and worsen cellular and tissue damage in the context of human disease.

### ATF6 activity is protective in 1-dSA-treated ROs

**Fig. 5:**
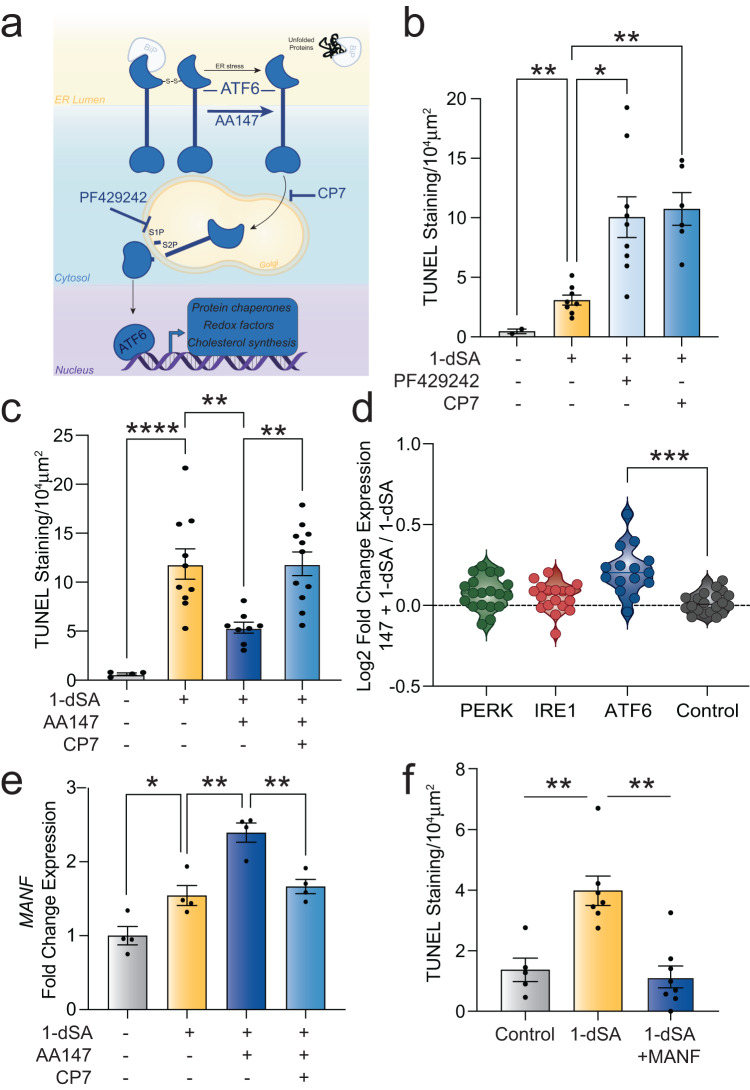
ATF6 promotes retinal cell protection in the context of 1-dSA toxicity. **a** Illustration of ATF6 arm of the UPR and pharmacologic modulators of this pathway. **b** Quantification of TUNEL staining of ROs treated with 1-dSA (1 µM) for 4 days in the presence or absence of Ceapin-A7 (CP7; 7 µM) or PF429242 (10 µM) presented as mean ± SEM. Dots represent biologically independent ROs tested concurrently. 1-dSA vs Control *p* = 0.0019, 1-dSA vs. PF429242 *p* = 0.0151, 1-dSA vs. CP7 *p* = 0.0077 for Welch ANOVA with Dunnett test for multiple comparisons to 1-dSA treatment. *p < 0.05, **p < 0.01. **c** Quantification of TUNEL staining of ROs treated with 1-dSA (1 µM) and/or AA147 (10 µM) ± CP7. Presented as mean ± SEM. Dots represent biologically independent ROs tested concurrently. Control vs. 1-dSA *p* = 0.0002, 1-dSA vs. 1-dSA+AA147 *p* = 0.0088, 1-dSA vs. 1-dsA+AA147 + CP7 *p* > 0.9999; 1-dSA+CP7 vs. 1-dsA+AA147 + CP7 *p* = 0.0009 for ordinary one-way ANOVA with Šídák’s test. **p < 0.01, ****p < 0.0001. **d** Quantification of RNAseq fold changes of gene targets of the UPR-induced transcription factors ATF4, XBP1s, and ATF6 in ROs treated with 1-dSA ± AA147 (10 µM) for 3 and 4 days (*n* = 5 replicates of pooled ROs per condition). ATF6 vs. control *p* = 0.0003 for Welch ANOVA test with Dunnett T3 corrections for multiple comparisons to control gene set. ***p < 0.001. **e** qRT-PCR for expression of *MANF* relative to vehicle in ROs treated with 1-dSA and/or AA147 ± CP7. Presented as mean ± SEM for 4 biologically independent samples. Control vs. 1-dSA *p* = 0.0333, 1-dSA vs. 1-dSA+AA147 *p* = 0.0014, 1-dSA+AA147 vs 1-dSA+AA147 + CP7 *p* = 0.0048, for ordinary one-way ANOVA with Šídák’s test for multiple comparisons. *p < 0.05, **p < 0.01. **f** Quantification of TUNEL staining of ROs treated with 1-dSA (1 µM*)* ± MANF (100 ng/µL) for 4 days presented as mean ± SEM. Dots represent biologically independent ROs tested concurrently. Control vs. 1-dSA *p* = 0.0053, 1-dSA vs. 1-dSA+MANF *p* = 0.0019 for Welch ANOVA test with Dunnett T3 corrections for multiple comparisons. **p < 0.01. Source data are provided as a Source Data file.

The IRE1 and ATF6 signaling arms of the UPR are generally associated with adaptive remodeling of cellular physiology in response to ER stress. This is primarily mediated through the activation of the transcription factors ATF6 (a cleaved product of full-length ATF6) and XBP1s (downstream of IRE1) (Fig. 5a and Fig. S5a, respectively). These transcription factors induce the expression of genes involved in numerous adaptive biological pathways including ER proteostasis maintenance, cellular metabolism, and redox regulation^28,51^. Our transcriptional profiling showed transient increases in the expression of ATF6, and to a lesser extent IRE1/XBP1s, target genes in ROs treated with 1-dSA for 3 days (Fig. 3b). Thus, we sought to define the specific contributions of these UPR signaling pathways in 1-dSA toxicity using pharmacologic inhibitors and activators of these adaptive signaling pathways.

IRE1 was inhibited using compounds 4µ8c and STF-083010, both compounds that directly inhibit IRE1 RNAse activity required for IRE1-dependent XBP1s activation (Fig. S5a)^52,53^. We confirmed that co-treatment with these compounds inhibited 1-dSA-dependent increases in *XBP1s* (Fig. S5b). 1-dSA co-treatment with the IRE1 inhibitor STF-083010 increased toxicity in 1-dSA-treated cells; however, we did not observe similar increases upon co-treatment with 4µ8c (Fig. S5c). Further, pharmacologic activation of IRE1/XBP1s signaling using the IRE1/XBP1s activator compound IXA4^54^ upregulated XBP1s levels (Fig. S5d) but did not reduce toxicity in 1-dSA-treated retinal organoids (Fig. S5e). Collectively, these results suggest that IRE1/XBP1s activity is not prominently involved in influencing 1-dSA toxicity in ROs.

Next, we used pharmacologic activators and inhibitors of ATF6 to probe the importance of this pathway in 1-dSA retinal toxicity. ATF6 activity was inhibited using two compounds, Ceapin-A7 and PF429242, that block ATF6 activity through two distinct mechanisms (Fig. 5a). Ceapin-A7 inhibits ATF6 activation by preventing its trafficking to the Golgi for proteolytic activation^55^. In contrast, PF429242 inhibits site-1 protease (S1P) proteolytic activity required for release of the active, cleaved ATF6 transcription factor^56^. We confirmed that co-treatment with either Ceapin-A7 or PF429242 inhibited 1-dSA-dependent induction of the ATF6 target gene *HSPA5/BiP*, confirming the activity of these compounds in ROs (Fig. S6a). We also observed that co-treatment with either of these two ATF6 inhibitor increased toxicity in 1-dSA-treated ROs (Fig. 5b, Fig. S6b, c), suggesting that 1-dSA-induced ATF6 activation protects against cell death.

To further probe the contributions of ATF6 activation in 1-dSA-induced toxicity, we employed AA147—a pharmacologic activator of ATF6 transcriptional activity (Fig. 5a)^57^. AA147 promotes ATF6 activation through a mechanism involving increased trafficking to the Golgi for proteolytic activation^58^. We confirmed that co-treatment with AA147 increased the expression of the ATF6 target gene *HSPA5*/*BiP* in the presence of 1-dSA (Fig. S6d). Co-treatment with the ATF6 inhibitor Ceapin-A7 blocked AA147-dependent increases in *HSPA5/BiP* expression, confirming this compound increased *BiP* expression through an ATF6-dependent mechanism. AA147 treatment increased the survival of ROs challenged with 1-dSA (Fig. 5c, Fig. S6e). This protection was lost upon co-treatment with Ceapin-A7, indicating that AA147 increased protection through an ATF6-dependent mechanism.

Bulk RNAseq profiling of ROs treated with AA147 and 1-dSA showed increased expression of multiple ATF6 target genes (Fig. 5d, Supp. Data 6). IRE1/XBP1s target genes were not induced by AA147, reflecting the selectivity of this compound for ATF6 activation (Fig. 5d)^57^. However, PERK target genes were not significantly altered in AA147-treated cells co-treated with 1-dSA. This indicates that AA147-dependent ATF6 activation did not reduce 1-dSA toxicity by suppressing PERK/ISR signaling through a mechanism such as reductions of ER stress.

ATF6 regulates the expression of multiple genes known to be protective in the retina, including the secreted neurotrophic factor MANF—a protein primarily expressed in Müller glia (Fig. S7a)^59^. Extracellular MANF has previously been shown to protect the retina from diverse types of insults^58–62^. AA147 increases the expression of *MANF* above 1-dSA alone (Fig. 5e, S7b, c). AA147-induced *MANF* expression is suppressed by co-treatment with Ceapin-A7, confirming that AA147 increases *MANF* levels through an ATF6-dependent mechanism. This indicates that ATF6-dependent increases in MANF could contribute to the protection observed upon treatment with AA147. Consistent with this, the administration of recombinant MANF reduces 1-dSA-induced toxicity in ROs (Fig. 5f, Fig. S7d, e). These results suggest that AA147-dependent ATF6 activation protects against 1-dSA-induced toxicity through the upregulation of adaptive, protective target genes such as MANF.

## Discussion

The accumulation of cytotoxic 1-dSLs is linked to multiple retinopathies and neuropathies^3–6^, however, the mechanisms by which 1-dSLs impact retinal cell function are poorly understood. Here, we used multiple transcriptomic approaches to profile ROs treated with 1-dSA across time. We characterized a broad cellular response with a pronounced activation of the ER stress-activated UPR and subsequently validated the functional role of the UPR in 1-dSL toxicity by utilizing compounds that selectively inhibit or activate individual UPR signaling arms. This approach showed that PERK/ISR signaling mediates cell death through activation of pro-apoptotic and inflammatory pathways, whereas ATF6 promotes cell survival through mechanisms including upregulation of the secreted neurotrophic factor MANF. Although the activation of the pro-apoptotic PERK arm of the UPR is maintained throughout the 1-dSA treatment paradigm, we observed that activation of the pro-survival ATF6 arm is activated primarily at early stages prior to elevated cell death. Intriguingly, pharmacologic activation of ATF6 mitigates 1-dSA retinal toxicity without impacting PERK/ISR signaling. This indicates that imbalanced signaling through these pro-apoptotic and adaptive signaling arms of the UPR, and not simply sustained PERK/ISR signaling, is an important contributor to 1-dSL-induced retinal toxicity.

Our results suggest that enhancing/prolonging ATF6 signaling offers a unique opportunity to mitigate pathologic photoreceptor death induced by 1-dSA treatment. Pharmacologic inhibition of ATF6 exacerbates 1-dSL toxicity, demonstrating an adaptive, protective role for ATF6 signaling in the context of 1-dSL toxicity. Pharmacologically enhancing ATF6 activity with AA147 attenuated 1-dSL toxicity, indicating that increasing and maintaining signaling through this adaptive pathway could mitigate the toxicity associated with the disease. As AA147 did not reduce 1-dSA-mediated increases in inflammatory or apoptotic signaling pathways, this suggests that ATF6 activation may promote resilience to 1-dSA-induced stress rather than diminishing its toxicity.

ATF6 signaling is well established to be important for regulating retinal development and health. Hypomorphic mutations in *ATF6* impair cone photoreceptor differentiation in the disease achromatopsia^31^. As we observed, pharmacologic ATF6 activation can rescue this deficiency and restore cone photoreceptors in iPSC models of this disease^63^. Alternatively, deficiencies in ATF6 activation induced by environmental insults or aging contribute to retinal degeneration associated with other diseases including retinitis pigmentosa and cone-rod dystrophy^33,64^. The protection afforded by ATF6 in the retina is likely mediated through its regulation of multiple adaptive genes including the ER chaperones BiP and the neurotrophic factor MANF^28,65,66^. Each of these ATF6-regulated genes has been shown to protect photoreceptors and/or Müller glia against diverse types of insults including ER stress-induced apoptosis^33,67,59^, oxidative stress^68^, and age-related retinal inflammation^62^. We show that the exogenous addition of MANF alone is sufficient to rescue cell death in 1-dSL toxicity, indicating a likely role for ATF6-dependent regulation of *MANF* expression in the protection observed for AA147 and providing an additional prospective treatment to mitigate 1-dSL-associated retinal toxicity. However, it is important to note that AA147-dependent ATF6 activation likely mediates its protection through the regulation of multiple adaptive factors, underscoring the unique potential for pharmacologically targeting this UPR signaling pathway to mitigate 1-dSL toxicity in complex tissues such as the retina.

Pharmacologic inhibition of PERK kinase activity with GSK2656157 reduced 1-dSL toxicity, whereas inhibition of eIF2α signaling downstream of PERK using the compound ISRIB provided more substantial protection. This suggests that other eIF2α kinases of the ISR, apart from PERK, may also be involved in 1-dSA-mediated toxicity. Consistent with this, serine deprivation and exogenous 1-dSA addition can activate ISR kinases such as GCN2 and PKR that promote eIF2α phosphorylation and ATF4 transcriptional activity^69,70^. As ATF4 regulates the expression of serine synthesis genes, 1-dSL-induced activation of PERK and other eIF2α kinases may function as an adaptive response for regulating intracellular serine levels. However, since PERK/ISR signaling is important for regulating serine synthesis, pharmacologic targeting of these pathways for 1-dSL-associated disorders such as MacTel is unlikely to be a promising path forward for treating patients, as downregulation of serine synthesis genes would exacerbate the underlying pathology of elevated 1-dSLs.

Using snRNAseq, we show that within retinal tissue there are distinct cell-specific responses to 1-dSL, with photoreceptors and Müller glia demonstrating the most pronounced response. This is consistent with the pathogenesis of MacTel where these cellular subtypes are also the retinal cell types most profoundly impacted in this disease^71^. It remains unclear why photoreceptors and Müller glia are uniquely reactive to 1-dSLs. One possibility is an increased conversion of 1-dSA to the toxic 1-dhCER in photoreceptors and Müller glia through differential expression of CERS family enzymes. However, we do not observe higher expression of total CERS in photoreceptors and Müller glia, nor do we observe cell-specific expression of CERS family isoforms that correspond to toxicity. Another hypothesis is that cell-specific toxicity is dependent on the unique physiological demands of each cell type. If so, future work determining the physiological consequences of elevated 1-dSLs will be essential to understand the genesis of organelle dysfunction. Regardless, our analysis here suggests that the disruption of the ER in both photoreceptors and Müller glia is a prime event in 1-dSL toxicity in the retina.

Collectively, our results indicate that imbalanced signaling through the pro-apoptotic PERK/ISR and adaptive ATF6 arms of the UPR in two key retinal cell types, Müller glia, and photoreceptors, is a contributing factor in dictating 1-dSA toxicity in ROs. This provides a framework to better understand how 1-dSA promotes toxicity of the retina and peripheral neurons in diverse diseases including MacTel, diabetes, and HSAN1. Further, our results identify pharmacologic enhancement of ATF6 activity and/or exogenous addition of the ATF6-regulated neurotrophic factor MANF as potential strategies to promote adaptive remodeling of the retina to mitigate pathology associated with increases of 1-dSLs.

## Methods

### Organoid generation and maintenance

*Stem cells:* hiPSC lines were derived from peripheral blood mononuclear cells from a female (donor #1) and male (donor #2). Reprogramming was performed using Sendai virus for reprogramming factor delivery. Donor #1 hiPSCs were derived by the Harvard iPS core facility and donor #2 hiPSCs were derived by the Salk iPSC core facility. All experiments were performed using donor #1, except for Fig. S7e, which used donor #2. All cell lines were obtained with verified normal karyotype and were contamination-free. hiPSC were maintained on Matrigel (BD Biosciences) coated plates with mTeSR+ medium (STEMCELL Technologies). Cells were passaged every 3–4 days at ~80% confluence. Colonies containing clearly visible differentiated cells were marked and mechanically removed before passaging.

Retinal organoids were differentiated from hiPSCs between passage 10 and 20. Retinal organoids were initiated and differentiated as previously described^72^. Embryoid bodies (EBs) were generated and cultured in Neural Induction Media containing DMEM/F12 (Gibco; cat 11330057) with 1% N2 supplement (Gibco; cat 17502048), 1% MEM NEAA (Gibco; cat 11140050), and 2 mg/mL heparin (STEMCELL Technologies; cat 07980) at 180 U/mg and then plated on day 7. On day 16, media was changed to Retinal Differentiation Media (RDM) containing 48% DMEM/F12 and 48% DMEM (Gibco; cat 11995073) supplemented with 2% B27 supplement without vitamin A (Gibco; cat 12587010), 1% MEM NEAA, and 1% Pen-Strep (Gibco; cat 15140122). On day 28, EBs were mechanically dissociated and transferred to rotating suspension culture. At week 8 of differentiation, while in suspension culture, the media was switched to RDM plus 10% FBS (Corning; cat 35-016-CV), 100 μM Taurine (Sigma; cat TO-625), and 2 mM Glutamax (Gibco; cat 35050061). At week 17, mature retinal organoids were removed from rotating suspension culture and transferred to the stationary suspension culture until they were assayed at later time points.

Fully mature retinal organoids were assayed between 26 and 30 weeks post differentiation. We used 26-week-old ROs for the dSA toxicity time course experiments (Fig. 1b, c). All subsequent toxicity/rescue assays measuring TUNEL were performed using organoids between 28–30 weeks old. Bulk RNAseq and snRNAseq experiments were performed using organoids at 30 weeks old.

### Cell culture treatments

Lyophilized lipids, 1-deoxysphinganine (Avanti; cat 860493) and sphinganine (Avanti; cat 860498), were resuspended to stock concentrations at 5 mM in EtOH and subsequently added to retinal organoid culture media at a concentration of 1 μM. For control conditions, equivalent amounts of EtOH were added to the media. ISRIB (Sigma; cat SML0843) was administered at 200 nM. GSK2656157 (Bio Vision; cat 9466) was administered at 500 nM. AA147 was obtained from the Kelly Lab at Scripps Research and resuspended in dimethyl sulfoxide (DMSO); organoids were administered at 10 μM daily. PF429242 (Sigma-Aldrich; cat SML0667) was resuspended in water and administered at 10 μM. CP7 was obtained from the Walter Lab at UCSF, resuspended in DMSO, and administered at 7 μM. 4μ8 C (EMD Millipore; cat 412512) and STF-083010 (Sigma; cat 412510) were resuspended in DMSO and administered at 32 µM. For drug experiments, 1-dSA and drugs were added to organoid culture media at concurrent times. Organoids were cultured in experimental conditions for 4 days unless otherwise stated with a condition-specific media change on day 2. For GSK2656157 and ISRIB experiments, drugs were added on day 0 and day 2. For all other drug experiments, drugs were added daily.

### Immunohistochemistry and TUNEL staining

Organoid tissue fixed in 4% PFA in PBS for 10 mins, washed in PBS, and then in 20% sucrose in PBS overnight. Tissues were embedded in the O.C.T compound and frozen. Cryosectioning was done at 12 µm and slices were mounted on glass polylysine-coated slides. Prior to primary antibodies samples were blocked with 5% donkey serum in PBS. Primary antibodies were added to samples at 4 °C and incubated overnight. Following primary antibodies samples were washed 3 × 10 mins in PBS. Secondary antibodies were added at room temperature for 2 h. Dapi was added at 1:1000 in PBS for 10 mins following secondary antibodies.

Primary antibodies: rabbit anti-Recoverin (1:500, Millipore AB5585), mouse anti Map2 (1:500, BD Bioscience 556320), rabbit anti ATF4 (1:200 Cell Signaling 11815) (Secondary antibodies: donkey anti-rabbit alexafluor 555 (1:1000, Invitrogen 31572), donkey anti rabbit alexafluor 488 (1:1000, Invitrogen 21206), donkey anti-mouse alexafluor 555 (1:1000, Invitrogen 31570), donkey anti mouse alexafluor 488 (1:1000, Invitrogen 21202). TUNEL staining was performed using In Situ Cell Death Detection Fluorecein kit (Sigma cat# 11684795910) prior to addition of anti-recoverin primary antibody. Each organoid was represented by one central cryostat slice. Overlapping TUNEL positive staining and DAPI staining within a recoverin-positive cell was counted as cell death within a photoreceptor. Cell death was normalized to the area of recoverin staining in the retinal organoid. A detailed protocol is demonstrated in Eade et al. 2021 JoVE.{Eade, 2021 #137}.

### Protein lysate preparation and immunoblotting

Cell lysates were prepared by lysing retinal organoids in RIPA buffer [50 mM 4-(2-hydroxyethyl)−1-piperazineethanesulfonic acid (HEPES) pH 7.5, 140 mM NaCl, 1% Triton X-100, 1 mM Ethylenediaminetetraacetic acid (EDTA), and protease inhibitor cocktail (Roche)]. The total protein concentration in cellular lysates was normalized using the Bradford protein assay. Lysates were denatured with 1× Laemmli buffer + 100 mM dithiothreitol (DTT) and boiled before being separated by SDS-PAGE. Samples were transferred onto nitrocellulose membranes (Bio-Rad). Membranes were then incubated overnight at 4 °C. mouse anti-α-Tubulin primary antibody (1:2000; Sigma-Aldrich T6074) and rabbit anti-MANF (1:1000, Proteintech 10869-1-AP). Membranes were washed in TBST, incubated with the species-appropriate IR-Dye conjugated secondary antibodies, and analyzed using the Odyssey Infrared Imaging System (LI-COR Biosciences). Quantification was carried out with LI-COR Image Studio software. Uncropped immunoblots are provided in the Source Data file.

### RNA isolation and quantitative reverse transcriptase PCR (qRT-PCR)

Total RNA was purified from frozen tissues using Trizol Reagent (Life Technologies) according to the manufacturer’s instructions. First-strand cDNA was synthesized from 400 ng of total RNA using High-Capacity Reverse Transcriptase kit (Applied Biosystems) according to the manufacturer’s instructions. Individual 10 μl SYBR Green Master Mix (Applied Biosystems) real-time PCR reactions consisted of 2 μl of diluted cDNA, 5 μl of Power Up SYBR Green(Applied Biosystems), and 1 μl of each 5 μM forward and reverse primers. The PCR was carried out on 384-well plates on a Quant Studio Real-Time PCR system(Applied Biosystems) using a three-stage program: 95 °C for 10 min, 40 cycles of 95 °C for 20 s, 60 °C for 20 s and 72 C for 20 s. PCR data for intergene comparison were corrected for primer efficiency. Samples were normalized to the internal loading control, 36B4.

Primers:

**Table:** 

Gene	Forward	Reverse
36B4	GAAGCCACGCTGCTGAACAT	CAAGGCCAGGACTCGTTTGTA
ATF3	CCTCTGCGCTGGAATCAGTC	TTCTTTCTCGTCGCCTCTTTTT
XBP1s	GCTGAGTCCGCAGCAGGT	CTGGGTCCAAGTTGTCCAGAAT
XBP1t	TGAAAAACAGAGTAGCAGCTCAGA	CCCAAGCGCTGTCTTAACTC
HSPA5	GCCTGTATTTCTAGACCTGCC	TTCATCTTGCCAGCCAGTTG
MANF	TTTACCAGGACCTCAAAGACAGA	TTGCTTCCCGGCAGAACTTTA

### Statistics and reproducibility

All experiments were performed with a minimum of two experimental replicates, with spatial and temporal separation of individual experiments to ensure robust validity of findings. The number of biological replicates and/or independent experiments presented in each figure panel are as depicted or as stated in the figure legends. Statistical analyses were performed using Prism 9 (GraphPad, San Diego, CA) as described. One-way ANOVA statistical tests were used to detect statistically significant differences between the means of three or more treatments with post hoc testing for multiple corrections as noted to define specific statistical relationships, except for cases in which data showed significantly different standard deviations using the Brown-Forsythe test; in these instances, comparisons were statistically tested using Welch ANOVAs. Two-way ANOVA tests were used to detect statistically significant changes in multiple genes across multiple conditions.

#### Nuclei isolation

For snRNAseq, nuclei were isolated following a modified version of 10X Genomics’ Nuclei Isolation from Mouse Brain Tissue for Single Cell ATAC Sequencing (CG000212, Rev. B), Protocol 2. 500 µl of chilled 0.1× Lysis Buffer (10 mM Tris-HCl pH 7.4, 10 mM NaCl, 3 mM MgCl2, 0.01% Nonidet P40 Substitute, 1% BSA, 0.2 U/µl Rnase Inhibitor) was added to a tube of retinal organoid tissue and triturated 15–20 times. The suspension was then transferred to a dounce homogenizer cylinder and homogenized 10 times with an A pestle, then 10 times with a B pestle. The suspension was transferred to a new tube and incubated on ice for 5 minutes. The suspension was then pipette-mixed 10 times, then incubated on ice for another 10 minutes. 500 µl chilled Wash Buffer (10 mM Tris-HCl pH 7.4, 10 mM NaCl, 3 mM MgCl_2_, 1% BSA, 0.2 U/µl Rnase Inhibitor) was then added, and the suspension was pipette-mixed five times and then passed through a 40 µm Flowmi Cell Strainer into a new tube. Nuclei concentration was determined using a Countess II FL Automated Cell Counter, then the nuclei were centrifuged at 500 × *g* for 5 min at 4 C. The supernatant was discarded and the nuclei were resuspended in the proper volume of chilled Diluted Nuclei Buffer (1× 10X Genomics Nuclei Buffer, 0.2 U/µl RNase Inhibitor) to achieve a concentration range of 700–1200 nuclei/µl, again in order to target 10,000 cells for sequencing. This recommended concentration was confirmed using Countess II, and then the 10× snRNAseq protocol was followed to completion.

### RNA-sequencing analysis

Whole transcriptome RNA sequencing was conducted at the Scripps Research Institute Genomics Core. Libraries were prepared using the NEB Ultra II and then sequenced on an Illumina NextSeq 2000 platform to a depth of 20 M 75 bp SE reads per condition. The DNAstar Lasergene Suite (including Seqman Ngen17 and Arraysstar 17, was used to align reads were aligned to the human genome GRCh38 assembly and generate read counts respectively. Differential expression and statistical significance calculations between conditions were assessed with DESeq2 v.1.34.0 in R. Functional gene set enrichment analysis (fGSEA) was performed using the fGSEA package v. 1.20.0 in R. The Hallmark gene set (v7.5.1) was downloaded from MsigDB. UPR gene sets analysis was previously established in^38^. Enrichplot package v. 1.14.2 was used to generate the gene set network map.

Single-nucleus RNA sequencing was performed at the Scripps Research Institute Genomics Core using 10X Genomics Chromium 3’ v. 3 library preparation protocol and then sequenced on an Illumina NextSeq 2000. Two replicates were performed per condition. FASTQ files were aligned to GRCH38, introns included, using 10X Genomics CellRanger v. 6.0. Quality control metrics were assessed (UMI number, gene number, and mitochondrial percentage) and cells outside a defined range of feature counts, and mitochondrial percentages were excluded from downstream analysis (see code for details). After filtering, an average of 13,542 cells were included in each sample with estimated cell counts per replicate ranging from 12,635—13,882. Seurat v. 4.0 SCTransform was used for normalization. Individual samples were integrated into a single dataset and Harmony v. 0.1 was used for refining sample integration. Canonical Correlation Analysis was used for cluster generation after which UMAP embedding was performed. Cluster cell identification was performed using markers previously identified in Thomas et al.^21^. Progenitor or immature classes were excluded from downstream analyses. The R package for Augur v.1.0.3 was downloaded from the neurorestore Github repository^37^ to perform cell type prioritization and to generate the UMAP of AUC for cluster responsiveness to 1-dSA treatment. DEG counts shown in Fig. 2c were generated using the FindAllMarkers command in Seurat with DESeq2 test used to compare 1-dSA treated organoids to control. Cluster-based fGSEA was performed on pseudobulk data from clusters with DESeq2 calculations for differentially expressed genes. Escape 1.0 was used for evaluating fGSEA activation at a single-nucleus resolution. The complete RNAseq data is deposited in gene expression omnibus (GEO) as GSE213948.

### Reporting summary

Further information on research design is available in the Nature Portfolio Reporting Summary linked to this article.

## Supplementary information

Supplementary Information

Supplementary Dataset 1

Supplementary Dataset 2

Supplementary Dataset 3

Supplementary Dataset 4

Supplementary Dataset 5

Supplementary Dataset 6

Reporting Summary

## Data Availability

The bulk RNAseq and snRNAseq raw data as well as bulk expression read counts generated in this study have been deposited in the GEO database under accession code GSE213948. Differential expression data generated in this study are provided in the Supplementary Information file. Uncropped immunoblots and raw numerical data presented in graphs are provided in the Source Data file. Any additional data are available from the authors upon request. Source data are provided with this paper.

## Source data

Source Data
Description of Additional Supplementary files
