# Supplementary material for: Imbalanced unfolded protein response signaling contributes to 1-deoxysphingolipid retinal toxicity
Source: Nat Commun. 2023 Jul 11;14:4119. doi: 10.1038/s41467-023-39775-w (PMC10336013; doi:10.1038/s41467-023-39775-w)
Supplement: Supplementary file 1 — Supplementary Information [file 41467_2023_39775_MOESM1_ESM.pdf]

Supplemental Information
Imbalanced Unfolded Protein Response Signaling Contributes to 1-Deoxysphingolipid Retinal Toxicity

Jessica D. Rosarda[1], Sarah Giles[1,2], Sarah Harkins-Perry[1,2], Elizabeth A Mills[1,2], Martin Friedlander[1,2], R. Luke Wiseman[1], Kevin T. Eade[1,2,*]

[1]Department of Molecular Medicine, The Scripps Research Institute, La Jolla, CA 92037
[2]Lowy Medical Research Institute, La Jolla, CA 92037
*To whom correspondences should be address: keade@lmri.net
Preprint available at BioRxiv: doi: https://doi.org/10.1101/2022.09.22.509071

Keywords: unfolded protein response; deoxysphingolipid; retinal degeneration; ATF6; PERK;

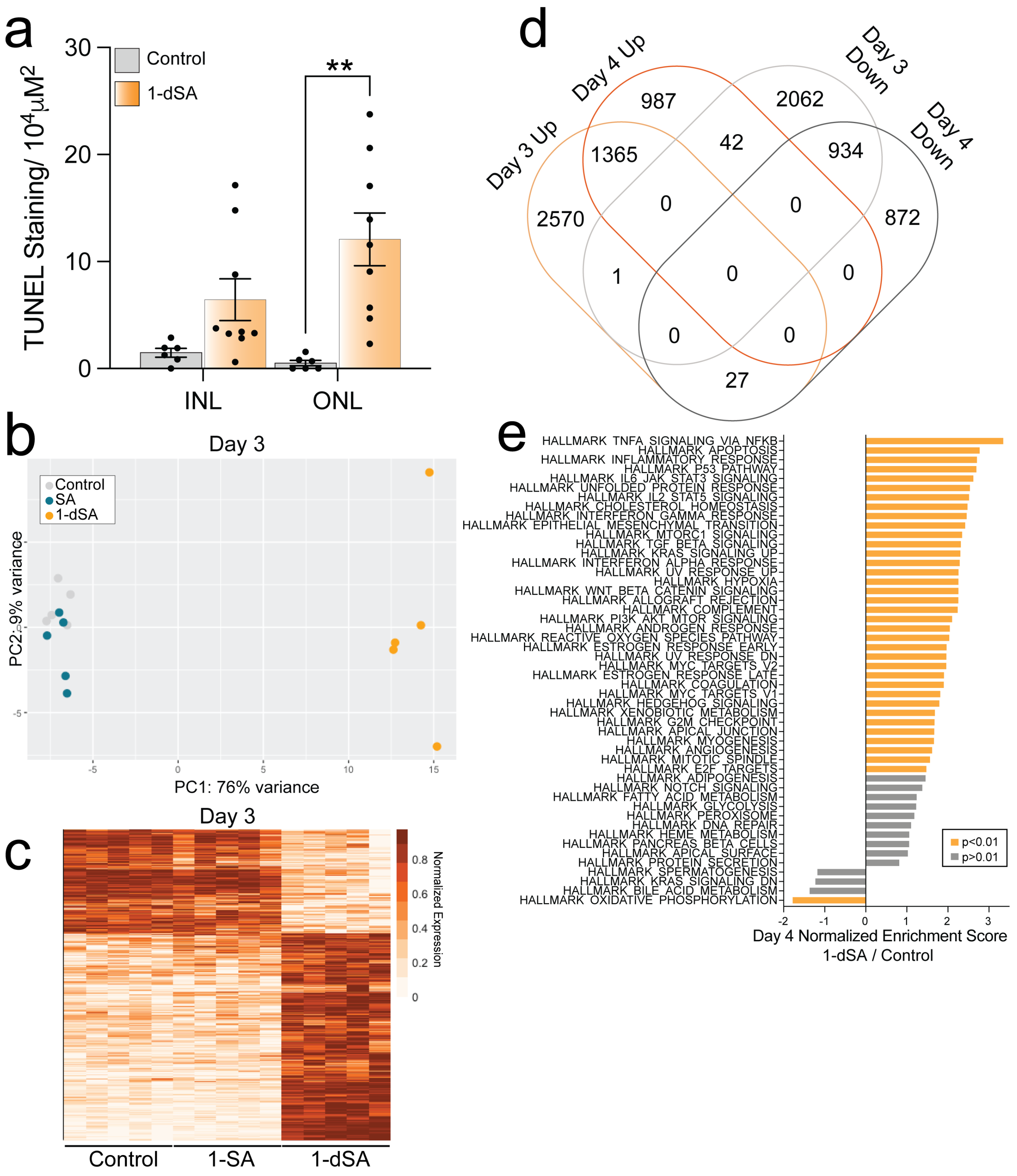

S1. 1-dSA-induced transcriptional remodeling precedes cell death. a. Quantification of TUNEL staining of ROs treated with 1-dSA (1μM) for 4 days in the inner nuclear layer (INL) or outer nuclear layer (ONL). Presented as mean +/- SEM. Dots represent biologically independent ROs tested concurrently. INL Control vs. 1-dSA p=0.062846, ONL Control vs. 1-dSA p=0.002326 for multiple unpaired, two-tailed t-tests between control and 1-dSA treated ROs. **p<0.01. **b**. Principal component (PC) analysis of all genes from bulk RNAseq data between

ROs treated for 3 days with vehicle, sphinganine (SA) (1μM), or 1-deoxysphinganine (1-dSA)(1μM). **c**. Heatmap comparison from bulk RNA-seq between ROs treated for 3 days with vehicle, sphinganine (SA) (1μM), or 1-deoxysphinganine (1-dSA)(1μM) for all genes with p-adj < 0.05 between 1-dSA and vehicle datasets. **d**. Venn diagram showing the overlap of genes increased or decreased in ROs treated for 3 or 4 days with 1-dSA (1 μM), as compared to control (n=5 replicates of 7 pooled ROs per condition). **e**. Enrichment of MsigDB Hallmark pathways in RNAseq data of ROs treated with 1-dSA relative to SA for 4 days (n=5 replicates of 7 pooled ROs per condition). Pathways with enrichment of p-adj < 0.01 are highlighted in orange. Source data are provided as a Source Data file.

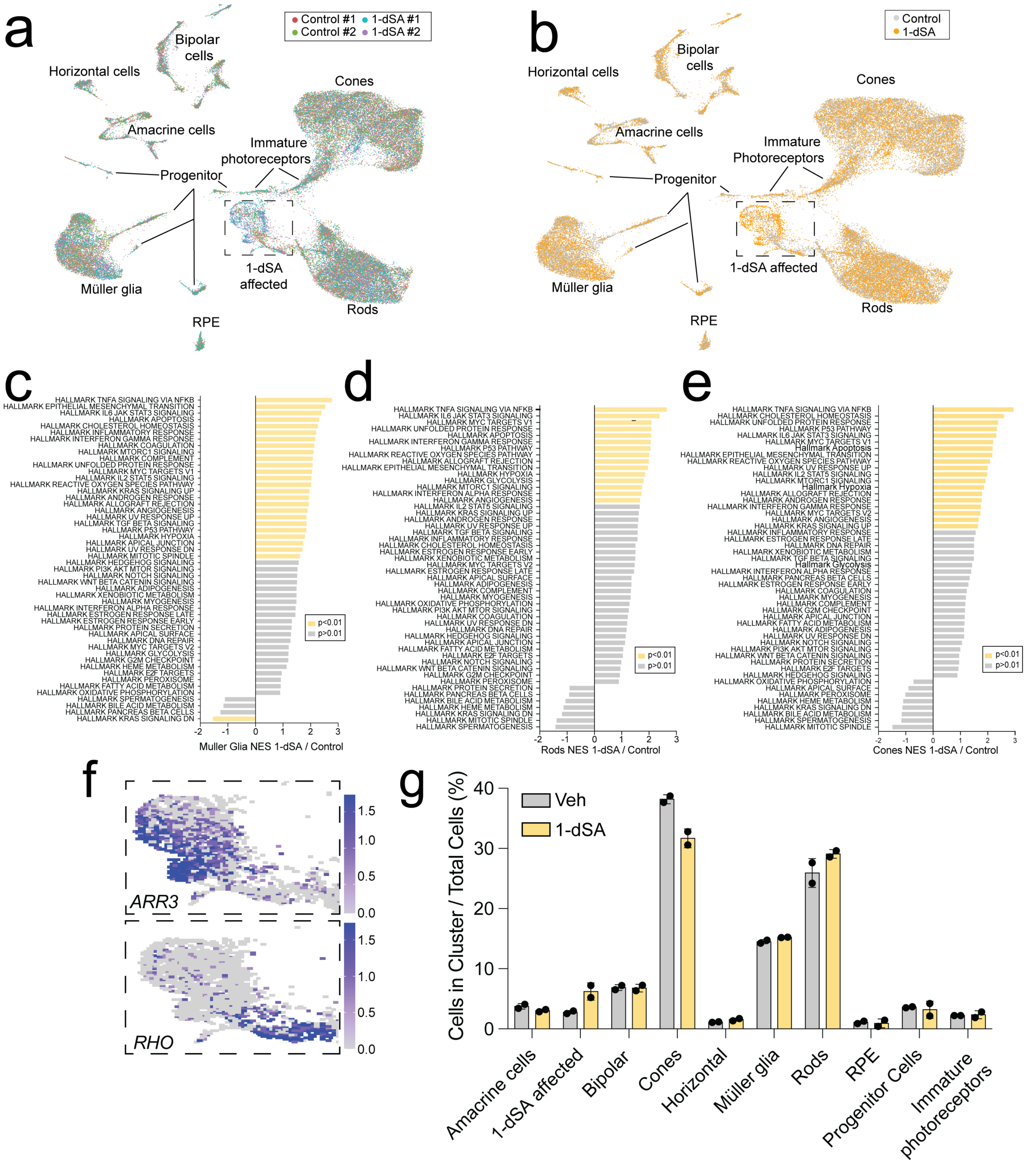

S2. Photoreceptors and Müller glia show a transcriptional response to 1-dSA. a. UMAP of unbiased clustering of snRNAseq data from pooled ROs treated with control or 1-dSA (1 μM) for 3 days by sample. **b.** UMAP of cells in snRNAseq data colored by treatment. 1-dSA-treated organoids are in orange; control-treated organoids are in grey. **c-e**. Enrichment of MsigDB Hallmark pathways in the Müller glia cluster **(c)**, rod cell cluster (**d**), and cone cell cluster (**e**) in control organoids relative to 1-dSA treated organoids (1 μM, 3 days). Pathways with enrichment of p-adj < 0.01 are highlighted in yellow. **f**. Expression of the cone marker *ARR3* and the rod

marker *RHO* in the 1-dSA affected cluster. **g**. Relative recovery of individual cell types from snRNAseq dataset of pooled ROs treated for 3 days with 1-dSA (1 µM, yellow) relative to control (grey) presented as mean +/- SD. Dots represent biologically independent pools of ROs tested concurrently. Source data are provided as a Source Data file.

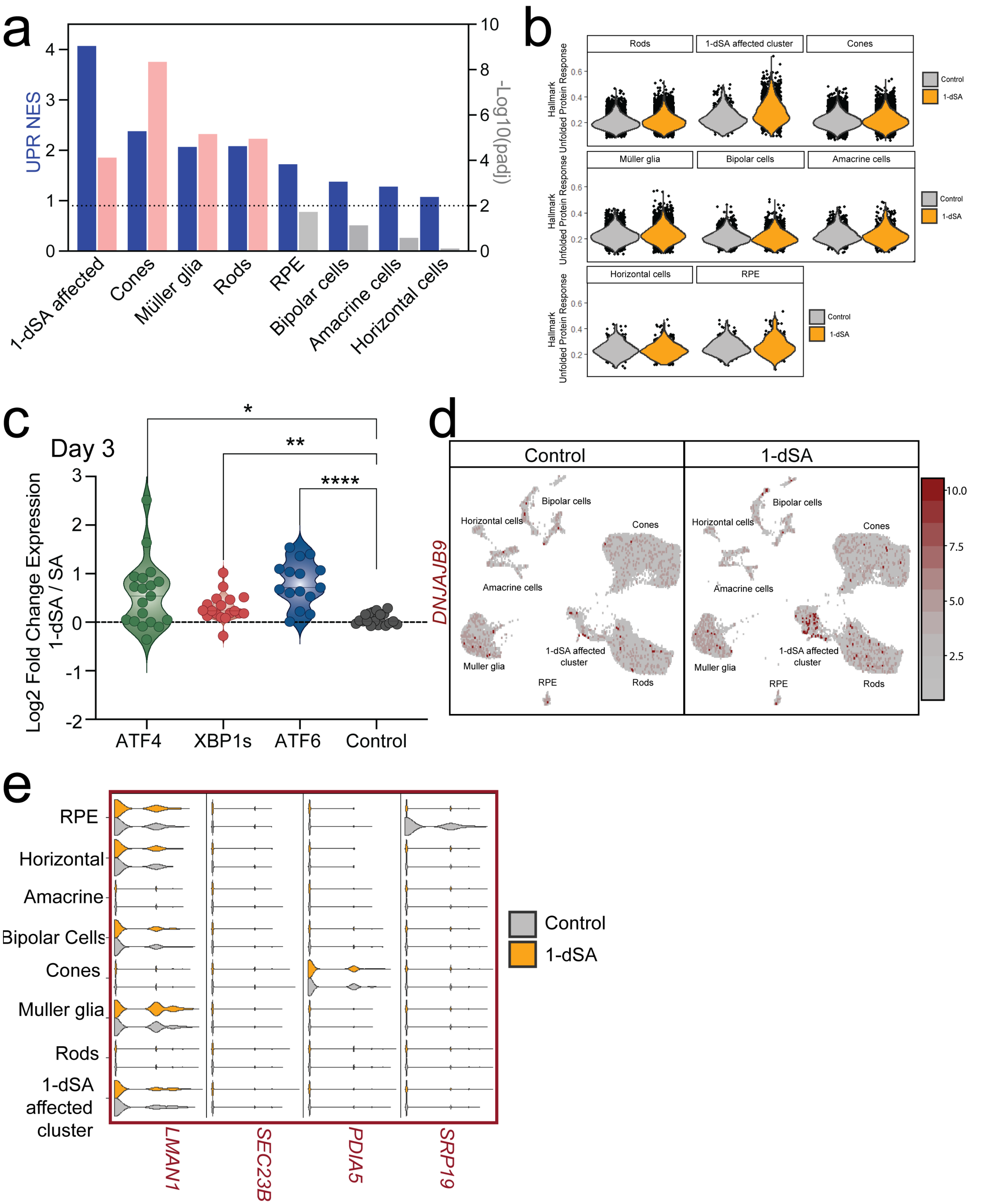
a
UPR NES
-Log10(padj)
1-dSA affected
Cones
Müller glia
Rods
RPE
Bipolar cells
Amacrine cells
Horizontal cells
b
Hallmark Unfolded Protein Response
Rods
1-dSA affected cluster
Cones
Müller glia
Bipolar cells
Amacrine cells
Horizontal cells
RPE
Control
1-dSA
c
Day 3
Log2 Fold Change Expression 1-dSA / SA
ATF4
XBP1s
ATF6
Control
*
**
****
d
Control
1-dSA
DNAJB9
Bipolar cells
Horizontal cells
Cones
Amacrine cells
1-dSA affected cluster
Muller glia
RPE
Rods
e
RPE
Horizontal
Amacrine
Bipolar Cells
Cones
Muller glia
Rods
1-dSA affected cluster
LMAN1
SEC23B
PDIA5
SRP19
Control
1-dSA

S3. The UPR is differentially activated in photoreceptors and Muller glia from 1-dSA treated organoids. **a**. MSigDB Hallmark fGSEA analysis for Unfolded Protein Response (UPR). Normalized enrichment scores (NES, blue) and -log10(pink: p-adj <0.01; grey: p-adj > 0.01) for 1-dSA treated cells relative to control cells from snRNAseq dataset are shown. **b**. Escape analysis of MSigDB Unfolded Protein Response (UPR) enrichment per cell in snRNAseq dataset from control (grey) or 1-dSA (1 µM, 3 days) treated organoids. **c**. Quantification of RNAseq fold changes of gene targets of the UPR-induced transcription factors ATF4, XBP1s, and ATF6 in ROs treated for 3 days with 1-dSA (1 µM) relative to those treated with sphinganine (SA, 1 µM). Control vs. ATF4 p=0.0163, Control vs. XBP1s p=0.0095, Control vs. ATF6 p < 0.0001 for Welch ANOVA test with Dunnett T3 corrections for multiple comparisons to control gene set. *p<0.05, **p<0.01, ****p<0.0001. **d.** Expression of the XBP1s target gene *DNAJB9* across mature cell types in control or 1-dSA (1 µM, 3 days) treated ROs. **e.** Violin plot of gene targets of XBP1s target genes from our snRNAseq dataset separated by cluster and treatment. 1-dSA-treated organoids (1 µM, 3 days) are in orange; control-treated organoids are in grey. Source data are provided as a Source Data file.

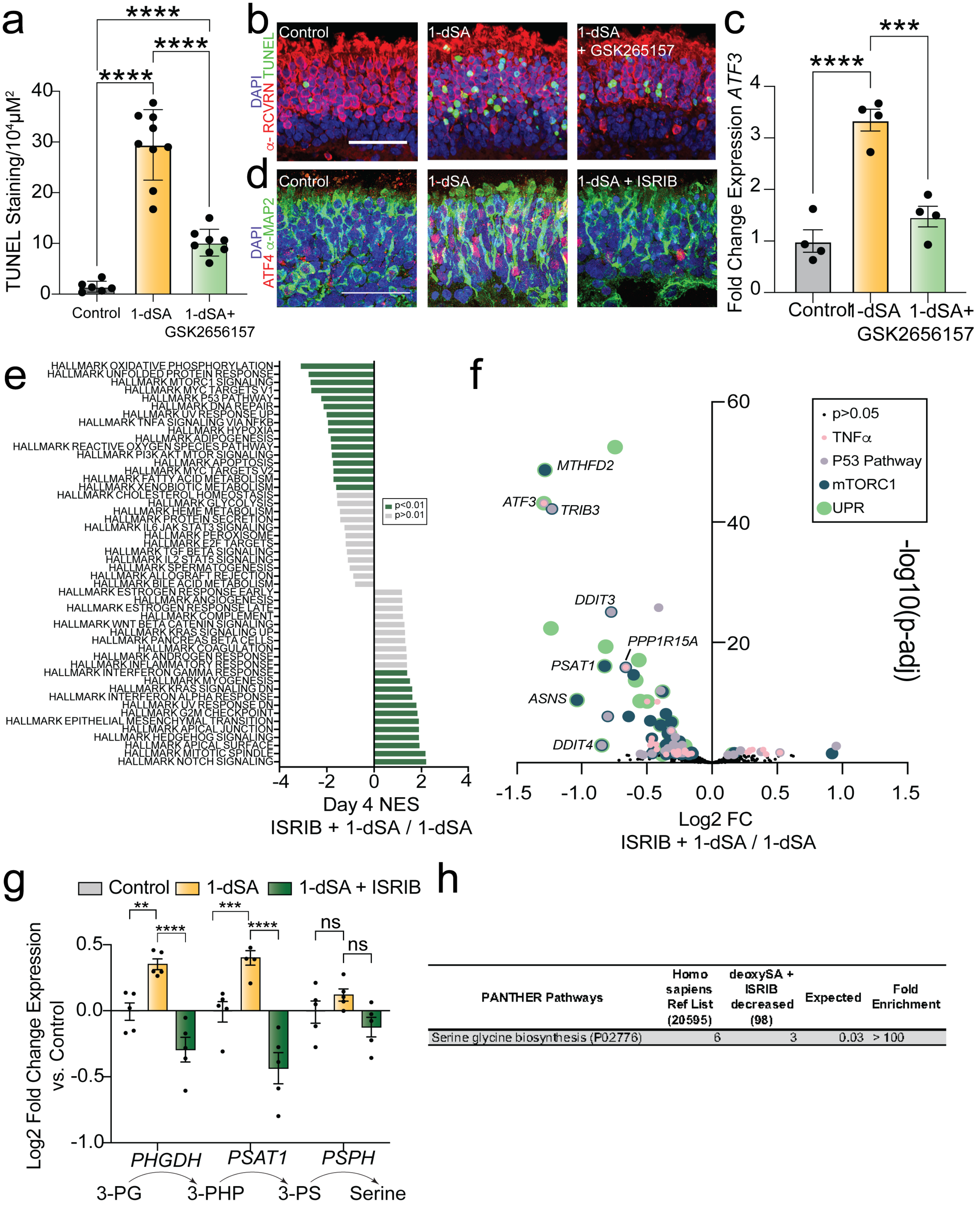

h

PANTHER Pathways	Homo sapiens Ref List (20595)	deoxySA + ISRIB decreased (98)	Expected	Fold Enrichment
Serine glycine biosynthesis (P02776)	6	3	0.03	> 100

S4. PERK/ISR signaling contributes to 1-dSA-induced retinal toxicity. a. qRT-PCR of the ATF4 gene target *ATF3* in biologically independent replicates of pooled ROs treated with vehicle, 1-dSA, or 1-dSA and GSK2656157. 1-dsA vs. Control p <0.0001, 1-dSA vs. 1-dSA+GSK2656157 p=0.0003 for ordinary one-way ANOVA with Dunnett corrections for multiple comparisons to 1-dSA. ***p<0.001, ****p<0.0001. **b,c**. Quantification and representative images of TUNEL staining of organoids treated for 4 days with 1-dSA (1 μM) ± GSK2656157 (500 nM). **b**. Quantification of TUNEL staining. Dots represent biologically independent ROs tested concurrently. p<0.0001 with Welch ANOVA test with Dunnett T3 corrections for multiple comparisons to 1-dSA. ****p<0.0001. **c.** Representative images of retinal organoids in (b) stained with nuclear DAPI (blue), α-recoverin (red), and TUNEL stain (green). Scale bar is 25μm. **d**. Representative images of retinal organoids treated with Control, 1-dSA (1 μM), and ISRIB (200 nM) for 4 days. Nuclear DAPI (blue), α-MAP2 (green), and α-ATF4 (red) are shown. Scale bar is 25μm. **e.** Enrichment of MSigDB Hallmark pathways in 1-dSA- and ISRIB-treated organoids relative to 1-dSA-treated organoids. Pathways with enrichment of p-adj < 0.01 are in green. **f**. Scatterplot of MsigDB pathway genes enriched in ROs treated with ISRIB+1-dSA relative to 1-dSA. Pathways that contain the same genes show overlapping markers. **g.** Expression fold changes of the genes *PHGDH*, *PSAT1*, and *PSPH* from pooled ROs treated with 1-dSA (1μM) ± ISRIB for 4 days compared to control-treated ROs. Each dot represents relative expression in an individual RNA-seq replicate. 3-PG=3-phosphoglycerate; 3-PHP=3-phosphohydroxypyruvate; 3-PS=3-phosphoserine. *PHGDH* 1-dSA vs. control p=0.0058, 1-dSA vs. 1-dSA+ISRIB p<0.0001, Control vs. 1-dSA+ISRIB p =0.0299; *PSAT1* 1-dSA vs. control p=0.0016, 1-dSA vs. 1-dSA+ISRIB p<0.0001, Control vs. 1-dSA+ISRIB p =0.0010; *PSPH* 1-dSA vs. control p=0.4625, 1-dSA vs. 1-dSA+ISRIB p=0.0764, Control vs. 1-dSA+ISRIB p=0.5494 for two-way ANOVA with Tukey's test for multiple comparisons to 1-dSA. **p<0.01, *** p<0.001, ****p<0.0001. **h**. Gene ontology analysis of Panther pathways for differentially expressed genes with p-adj<0.05 and Log2FC < -0.5 between ROs treated with ISRIB+1-dSA relative to 1-dSA-treated ROs. Bar charts used to present data as mean +/- SEM. Source data are provided as a Source Data file.

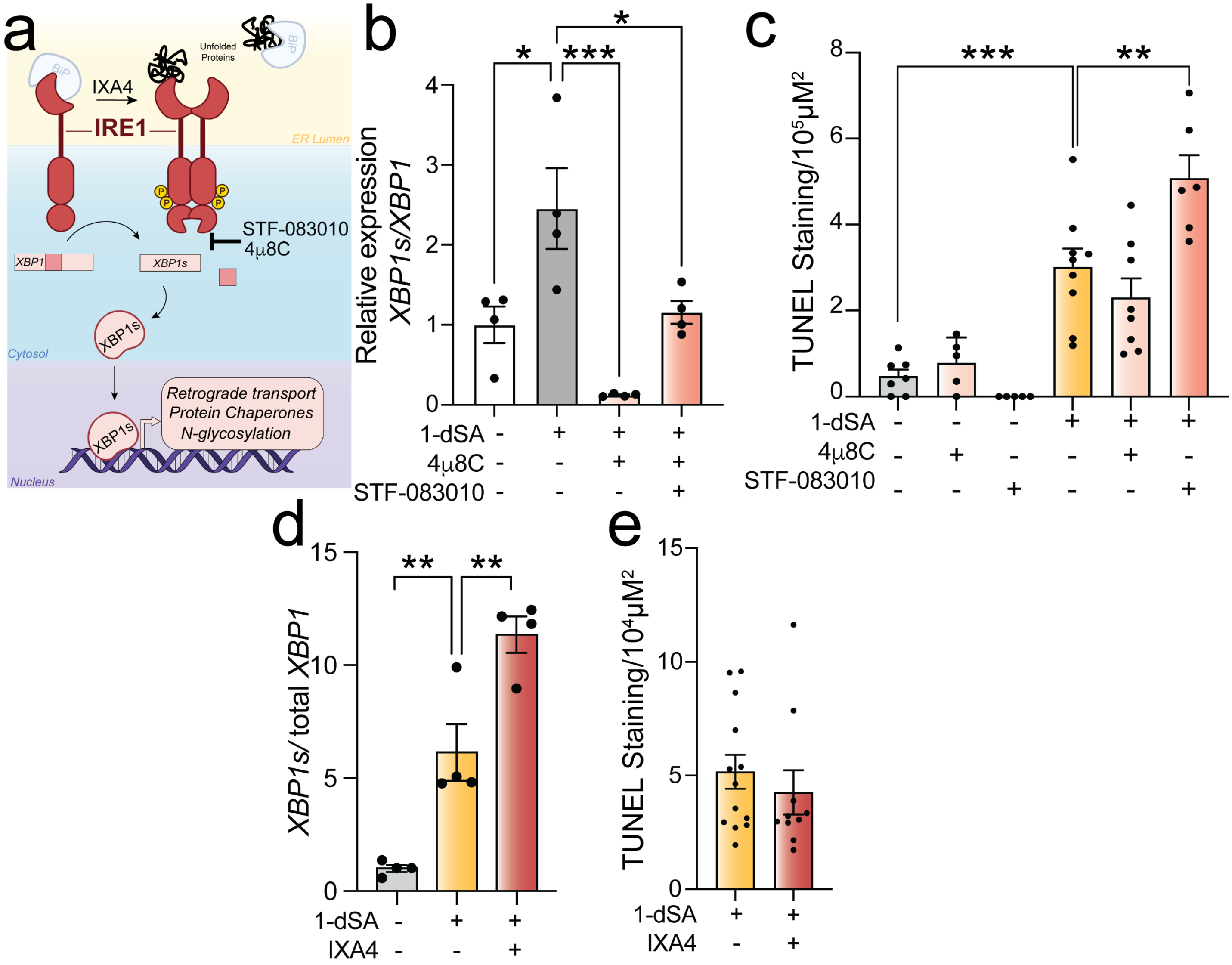

S5. IRE1 activity has limited impact on 1-dSA toxicity. **a**. Illustration of the IRE1/XBP1s UPR signaling pathway and pharmacologic inhibitors. **b**. Expression of spliced *XBP1* (*XBP1s*) relative to total *XBP1* ROs, measured using RT-qPCR, treated for 4 days with 1-dSA± IRE1 inhibitors 4μ8C (32 μM) or STF-083010 (32 μM). Control vs. 1-dSA p=0.0111, 1-dSA vs. 1-dSA+4μ8C p=0.0003, 1-dSA vs. 1-dSA+STF-083010 p=0.0227 for ordinary one-way ANOVA with Dunnett test for multiple comparisons to 1-dSA. Dots represent biologically independent replicates of pooled ROs. *p<0.05, **p<0.01, ***p<0.001 **c**. Quantification of TUNEL staining of ROs treated with 1-dSA (1μM) for four days ± 4μ8C (32 μM) or STF-083010 (32 μM). Dots represent biologically independent replicates of ROs tested concurrently. 1-dSA vs. Control p=0.0001, 1-dSA vs. 1-dSA+STF-083010 p=0.0029 for ordinary one-way ANOVA with Šídák's multiple comparisons test. Error bars show SEM. **p<0.01, ***p<0.001. **d.** Mean expression, measured with RT-qPCR, of spliced *XBP1* relative to total *XBP1* in pooled ROs treated for 4 days with 1-dSA (1 μM) ± the IRE1 activator IXA4 (10 μM). Control vs. 1-dSA p=0.0046, 1-dSA vs. 1-dSA vs. IXA4 p=0.0043 for ordinary one-way ANOVA with Šídák's multiple comparisons test. Error bars show SEM. Dots represent biologically independent replicates. **p<0.01. **e**. Quantification of TUNEL staining of ROs treated with 1-dSA (1μM) for four days ± IXA4 (10 μM). Dots represent biologically independent replicates of ROs tested concurrently. Bar charts present data as mean + SEM. Source data are provided as a source data file.

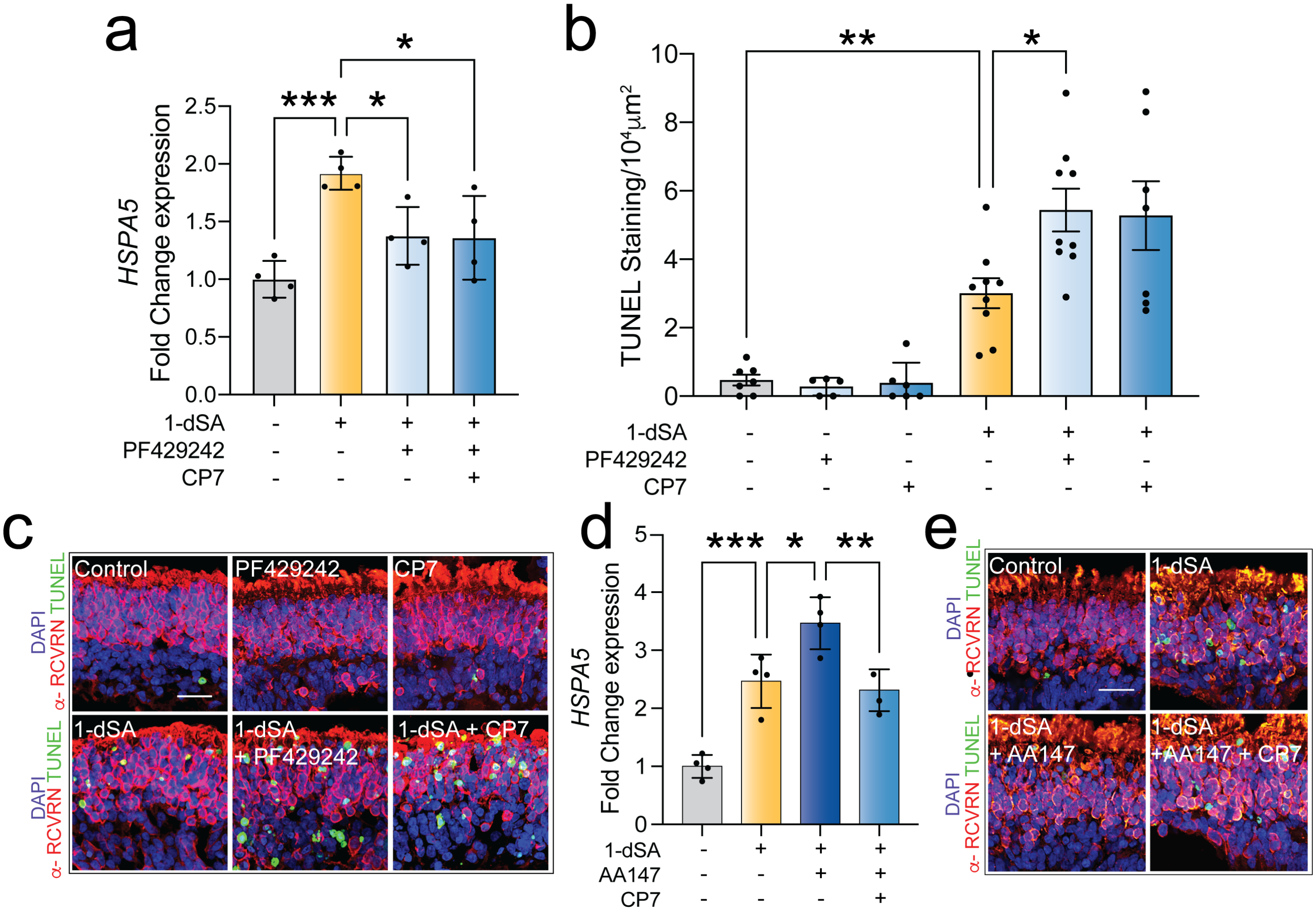

S6. ATF6 activity protects the retina from 1-dSA toxicity a. q-RTPCR of *HSPA5* expression in pooled ROs treated with 1-dSA (1 µM) ± Ceapin-A7 (CP7; 7 µM) or PF429242 (10 µM). Error bars show SEM. Dots represent biologically independent replicates. for ordinary one-way ANOVA with Šídák's multiple comparisons test. *p<0.05, ***p<0.001. **b**. Quantification of TUNEL staining of ROs treated with 1-dSA (1 µM) for 4 days ± ATF6 inhibitors Ceapin-A7 (CP7; 7 µM) or PF429242 (10 µM). Dots represent biologically independent replicates of ROs tested concurrently. 1-dSA vs. 1-dSA+PF429242 p=0.0305, for Welch ANOVA with Dunnett test for multiple comparisons to 1-dSA. *p<0.05, **p<0.01. **c.** Representative images of retinal organoids quantified in (**b**) treated with 1-dSA (1 µM), CP7 (7 µM), or PF-429242 (10 µM) for 4 days. DAPI staining, TUNEL staining, and α-recoverin (α-RCVRN) staining are shown. Scale bar is 25µm. **d.** qRT-PCR of mean expression of *HSPA5* relative to vehicle in ROs treated with 1-dSA (1 µM) ± AA147 (10 µM) and/or Ceapin-A7 (CP7; 7 µM). *p<0.05, **p<0.01, ***p<0.001 for ordinary one-way ANOVA with Šídák's multiple comparisons test. Dots represent biologically independent replicates of pooled ROs. **e**. Representative images of retinal organoids in quantified in (**Fig. 5c**) treated with 1-dSA (1 µM), AA147 (10 µM), or Ceapin-A7 (CP7; 7 µM) for 4 days. DAPI staining, TUNEL staining, and α-recoverin (α-RCVRN) staining are shown. Scale bar is 25µm. Bar charts present data as mean + SEM. Source data are provided as a Source Data file.

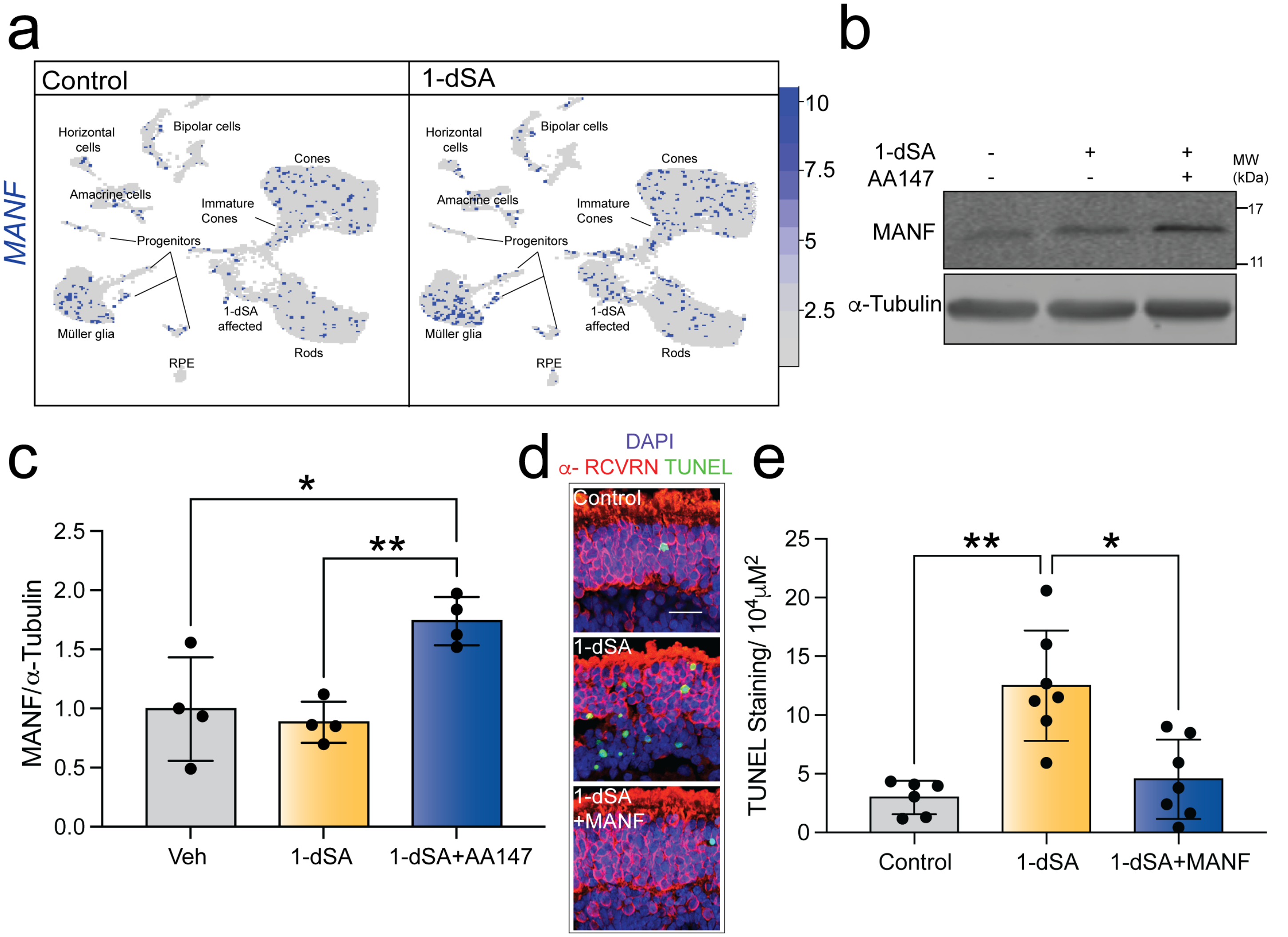

S7. *MANF* protects the retina from 1-dSA toxicity. a. Expression of *MANF* across cell types in ROs ± 1-dSA (1 μM; 3 days). **b**. Representative immunoblots showing MANF protein expression in lysates prepared from retinal organoids treated with 1-dSA (1μM) in the presence or absence of AA147 (10 μM) for 4 days. Tubulin is used as a loading control. **c.** Average MANF protein expression, relative to vehicle, in lysates prepared from pools of biologically independent retinal organoids treated with 1-dSA (1μM) in the presence or absence of AA147 (10 μM) for 4 days relative to α-Tubulin expression with four biologically independent replicates int. *$p<0.05$, **$p<0.01$ for ordinary one-way ANOVA with Tukey's correction for multiple comparisons between conditions. **d.** Representative images of retinal organoids quantified in (**Fig. 5f**) treated with 1-dSA (1 μM), and MANF (100 ng/μL) for 4 days. DAPI staining, TUNEL staining, and α-recoverin (α-RCVRN) staining are shown. Scale bar is 25μm. **e**. Quantification of TUNEL staining of ROs treated with 1-dSA (1 μM) ± MANF (100 ng/μL) for 4 days. Error bars show SEM. Dots represent biologically independent ROs tested concurrently. ** $p<0.01$ for Welch ANOVA test with Dunnett T3 corrections for multiple comparisons between conditions. Bar charts used to present data as mean + SEM. Source data are provided as a Source Data file.
