# Supplementary material for: Imbalanced unfolded protein response signaling contributes to 1-deoxysphingolipid retinal toxicity
Source: Nat Commun. 2023 Jul 11;14:4119. doi: 10.1038/s41467-023-39775-w (PMC10336013; doi:10.1038/s41467-023-39775-w)
Supplement: Supplementary file 8 — Reporting Summary [file 41467_2023_39775_MOESM8_ESM.pdf]

nature portfolio

Corresponding author(s): Kevin Eade

Last updated by author(s): Mar 8, 2023

Reporting Summary

Nature Portfolio wishes to improve the reproducibility of the work that we publish. This form provides structure for consistency and transparency in reporting. For further information on Nature Portfolio policies, see our Editorial Policies and the Editorial Policy Checklist.

Statistics

For all statistical analyses, confirm that the following items are present in the figure legend, table legend, main text, or Methods section.

n/a	Confirmed	
☐	☒	The exact sample size (*n*) for each experimental group/condition, given as a discrete number and unit of measurement
☐	☒	A statement on whether measurements were taken from distinct samples or whether the same sample was measured repeatedly
☐	☒	The statistical test(s) used AND whether they are one- or two-sided<br>*Only common tests should be described solely by name; describe more complex techniques in the Methods section.*
☐	☒	A description of all covariates tested
☐	☒	A description of any assumptions or corrections, such as tests of normality and adjustment for multiple comparisons
☐	☒	A full description of the statistical parameters including central tendency (e.g. means) or other basic estimates (e.g. regression coefficient) AND variation (e.g. standard deviation) or associated estimates of uncertainty (e.g. confidence intervals)
☐	☒	For null hypothesis testing, the test statistic (e.g. *F*, *t*, *r*) with confidence intervals, effect sizes, degrees of freedom and *P* value noted<br>*Give P values as exact values whenever suitable.*
☐	☒	For Bayesian analysis, information on the choice of priors and Markov chain Monte Carlo settings
☐	☒	For hierarchical and complex designs, identification of the appropriate level for tests and full reporting of outcomes
☒	☐	Estimates of effect sizes (e.g. Cohen's *d*, Pearson's *r*), indicating how they were calculated

Our web collection on statistics for biologists contains articles on many of the points above.

Software and code

Policy information about availability of computer code

Data collection	no software used for data collection
Data analysis	For RNA sequencing analysis, reads were aligned to the human genome GRCh38 assembly. For bulk RNA seq, this was performed using the DNAStar Lasergene 17 suite; for snRNAseq, 10X Genomics CellRanger v. 6.0 was used. R version 4.2.1 was used for Augur analysis and R version 4.0.3 was used for all other analyses. Differential expression and statistical significance calculations between conditions were assessed with DESeq2 v.1.34.0. Functional gene set enrichment analysis (fGSEA) was performed using the fGSEA package v. 1.20.0 in R. The Hallmark gene set (v7.5.1) was downloaded from MsigDB. UPR gene sets analysis were previously established in (39). Seurat v. 4.0 was used for single nucleus sequencing analysis. Enrichplot package v. 1.14.2 was used to generate the gene set network map. The R package for Augur v.1.0.3 was downloaded from the neurorestore Github repository.<br>All other statistical analyses were performed using Prizm 9 (GraphPad, San Diego, CA)

For manuscripts utilizing custom algorithms or software that are central to the research but not yet described in published literature, software must be made available to editors and reviewers. We strongly encourage code deposition in a community repository (e.g. GitHub). See the Nature Portfolio guidelines for submitting code & software for further information.

Data

Policy information about availability of data

All manuscripts must include a data availability statement. This statement should provide the following information, where applicable:

- Accession codes, unique identifiers, or web links for publicly available datasets
- A description of any restrictions on data availability
- For clinical datasets or third party data, please ensure that the statement adheres to our policy

All bulk RNAseq and scRNAseq raw data are deposited in gene expression omnibus (GEO) as GSE213948: https://www.ncbi.nlm.nih.gov/geo/query/acc.cgi?acc=GSE213948. Additionally, differential expression comparisons are provided as Supplementary Data in this manuscript.
Human genome reference GRCh38 (GENCODE v32/Ensembl 98) https://www.gencodegenes.org/human/
Hallmark Gene Set (MSigDB): https://www.gsea-msigdb.org/gsea/

Human research participants

Policy information about studies involving human research participants and Sex and Gender in Research.

Reporting on sex and gender	N/A
Population characteristics	N/A
Recruitment	N/A
Ethics oversight	N/A

Note that full information on the approval of the study protocol must also be provided in the manuscript.

Field-specific reporting

Please select the one below that is the best fit for your research. If you are not sure, read the appropriate sections before making your selection.

☒ Life sciences ☐ Behavioural & social sciences ☐ Ecological, evolutionary & environmental sciences

For a reference copy of the document with all sections, see nature.com/documents/nr-reporting-summary-flat.pdf

Life sciences study design

All studies must disclose on these points even when the disclosure is negative.

Sample size	sample size was determined by selecting the largest sample size feasible for the experiment
Data exclusions	For snRNAseq cells outside a defined range of feature counts and mitochondrial percentages were excluded from downstream analysis (see code for details). parameters were not predetermined
Replication	all replicates are stated in the text of the manuscript
Randomization	allocation was random
Blinding	TUNEL assays were analyzed using blinding. For all transcriptomic sequencing experiments, people performing sequencing were blinded to treatment groups.

Reporting for specific materials, systems and methods

We require information from authors about some types of materials, experimental systems and methods used in many studies. Here, indicate whether each material, system or method listed is relevant to your study. If you are not sure if a list item applies to your research, read the appropriate section before selecting a response.

Materials & experimental systems

n/a	Involved in the study
☐	☒ Antibodies
☐	☒ Eukaryotic cell lines
☒	☐ Palaeontology and archaeology
☒	☐ Animals and other organisms
☒	☐ Clinical data
☒	☐ Dual use research of concern

Methods

n/a	Involved in the study
☒	☐ ChIP-seq
☒	☐ Flow cytometry
☒	☐ MRI-based neuroimaging

Antibodies

Antibodies used	mouse anti alpha-Tubulin primary antibody (1:2000; Sigma-Aldrich T6074), rabbit anti MANF (1:1000, Proteintech 10869-1-AP), rabbit anti Recoverin (1:500, Millipore AB5585), mouse anti Map2 (1:500, BD Bioscience 556320), rabbit anti ATF4 (1:200 Cell Signaling 11815) (Secondary antibodies: donkey anti rabbit alexafluor 555 (1:1000, Invitrogen 31572), donkey anti rabbit alexafluor 488 (1:1000, Invitrogen 21206), donkey anti mouse alexafluor 555 (1:1000, Invitrogen 31570), donkey anti mouse alexafluor 488 (1:1000, Invitrogen 21202)
Validation	All antibodies were tested and validated by supplier. validation provided on product sheet of antibodies

Eukaryotic cell lines

Policy information about cell lines and Sex and Gender in Research

Cell line source(s)	iPSCs derived from human female PBMCs were derived by authors
Authentication	cell line was karyotyped
Mycoplasma contamination	cell lines were tested for mycoplasm and confirmed negative
Commonly misidentified lines (See ICLAC register)	no commonly misidentified cell lines were used
