# Supplementary material for: Imbalanced unfolded protein response signaling contributes to 1-deoxysphingolipid retinal toxicity
Source: Nat Commun. 2023 Jul 11;14:4119. doi: 10.1038/s41467-023-39775-w (PMC10336013; doi:10.1038/s41467-023-39775-w)
Supplement: Supplementary file 10 — Description of Additional Supplementary files [file 41467_2023_39775_MOESM10_ESM.pdf]

Description of Additional Supplementary files

File name: Supplementary Data 1.

Description: RNAseq of human iPSC-derived retinal organoids treated with 1-dSA for 2 days.
The included excel spread shows the DESEQ2 analysis of RNAseq of retinal organoids treated with vehicle or 1-dSA (1 μM) for 2 days. The complete RNAseq data is deposited in gene expression omnibus (GEO) as GSE213948.

File name:Supplementary Data 2.

Description:RNAseq of human iPSC-derived retinal organoids treated with 1-SA or 1-dSA for 3 days.
The included excel spread shows the DESEQ2 comparisons of RNAseq data from human iPSC-derived retinal organoids treated with 1-SA (1 μM), 1-dSA (1 μM), or vehicle equivalent for 3 days. The complete RNAseq data is dep osited in gene expression omnibus (GEO) as GSE213948.

File name:Supplementary Data 3.

Description:RNAseq of human iPSC-derived retinal organoids treated with 1-dSA for 4 days.
The included excel spread shows the DESEQ2 comparisons of RNAseq data from human iPSC-derived retinal organoids treated with 1-dSA (1 μM) or vehicle equivalent. The complete RNAseq data is deposited in gene expression omnibus (GEO) as GSE213948.

File name:Supplementary Data 4.

Description:snRNAseq of human iPSC-derived retinal organoids treated with 1-dSA for 3 days.
The included excel spread shows the DESEQ2 comparisons of pseudobulk snRNAseq data from human iPSC-derived retinal organoids treated with 1-dSA (1 μM) relative to vehicle equivalent. Clusters are labeled as noted in **Fig. 2A.** The complete RNAseq data is deposited in gene expression omnibus (GEO) as GSE213948.

File name:Supplementary Data 5.

Description:RNAseq of human iPSC-derived retinal organoids treated with 1-dSA for 4 days in the presence or abse nce of ISRIB. The included excel spread shows the DESEQ2 comparisons of RNAseq data from human iPSC-derived retinal organoids treated with 1-dSA (1 μM) in the presence or absence of ISRIB (200 nM). Comparisons are as noted in the excel file. The complete RNAseq data is deposited in gene expression omnibus (GEO) as GSE213948.

File name:Supplementary Data 6.

Description:RNAseq of human iPSC-derived retinal organoids treated with 1-dSA for 4 days in the presence or absence of AA147. The included excel spread shows the DESEQ2 comparisons of RNAseq data from human iPSC-derived retinal organoids treated with 1-dSA (1 μM) in the presence or absence of AA147 (10 μM). Comparisons are as noted in the excel file. The complete RNAseq data is deposited in gene expression omnibus (GEO) as GSE213948.
